# IL-17a promotes hepatocellular carcinoma by increasing FAP expression in hepatic stellate cells via activation of the STAT3 signaling pathway

**DOI:** 10.1038/s41420-024-01995-4

**Published:** 2024-05-13

**Authors:** Dapeng Sun, Wen Li, Dongyang Ding, Kunjiang Tan, Wenbin Ding, Zongyan Wang, Siyuan Fu, Guojun Hou, Wei-ping Zhou, Fangming Gu

**Affiliations:** 1 The Third Department of Hepatic Surgery, Eastern Hepatobiliary Surgery Hospital, Naval Medical University, 225 Changhai Road, Shanghai, 200438 China; 2 The International Cooperation Laboratory on Signal Transduction, Eastern Hepatobiliary Surgery Hospital, Naval Medical University, Shanghai, 200438 China

**Keywords:** Gastrointestinal cancer, Transcription, Cell signalling

## Abstract

Studies have shown that hepatic stellate cells (HSCs) and interleukin-17a (IL-17a) play important roles in liver tumorigenesis. In addition, fibroblast activation protein-α (FAP) has been shown to be a key regulator of hepatic stellate cell activation. In this study, in vivo and in vitro experiments were performed to verify the promoting effects of IL-17a administration, IL-17a overexpression, and FAP upregulation in HSCs on liver fibrosis and liver tumorigenesis. The cleavage under targets & release using nuclease (CUT&RUN) technique was used to verify the binding status of STAT3 to the FAP promoter. The in vitro studies showed that IL-17a activated HSCs and promoted HCC development and progression. FAP and IL-17a overexpression also activated HSCs, promoted HCC cell proliferation and migration, and inhibited HCC cell apoptosis. The in vivo studies suggested that IL-17a and FAP overexpression in HSCs facilitated liver tumor development and progression. The CUT&RUN results indicated that FAP expression was regulated by STAT3, which could bind to the FAP promoter region and regulate its transcription status. We concluded that IL-17a promoted HCC by increasing FAP expression in HSCs via activation of the STAT3 signaling pathway.

## Introduction

Among malignant tumors, hepatocellular carcinoma (HCC) has a high incidence rate [1, 2]. Currently, recurrence and metastasis remain obstacles to successful treatment [3]. There is an urgent need to explore and elucidate the mechanisms of metastasis and recurrence to allow more accurate prediction of patient prognosis and the development of novel methods for intervention and treatment. Hepatic stellate cells (HSCs) is an important type of nonparenchymal cells in the liver cancer microenvironment [4]. Previous studies have confirmed that activation of HSCs is a key alteration occurring during the development of liver fibrosis [5]. At present, an increasing number of studies have found that activated HSCs in the inflammatory tumor microenvironment are closely related to the occurrence and development of tumors [6–10]. aHSCs can promote the growth, proliferation and invasion of tumor cells, thus playing an important role in the hepatic “inflammation-fibrosis-cancer” axis [11]. HSCs are not only the target cells but also the effector cells of liver inflammation. However, the specific mechanisms of the interactions between HSCs and tumor cells are unclear.

Interleukin-17 (IL-17) positive cells are enriched in many solid tumors, including liver tumors [12]. It is publicly acknowledged that IL-17 can mediate inflammatory responses, promote angiogenesis and tissue remodeling, facilitate the clearance of exogenous pathogens, and restore normal tissue architecture after acute injury. However, these activities might also generate an inflammatory environment that in turn promotes tumorigenesis [13]. Studies have shown that IL-17-related signaling pathways can mediate neutrophil recruitment [14], facilitate tumor angiogenesis [15], and increase tumor invasiveness during the inflammatory response. In addition, IL-17a can also facilitate liver fibrosis by promoting HSCs activation, an observation suggesting the at least partially essential role of IL-17a in the occurrence of HCC.

Fibroblast activation protein-α (FAP) is selectively expressed in interstitial fibroblasts in more than 90% of malignant epithelial tumors, such as breast cancer [16, 17], ovarian cancer [18, 19], gastric cancer [20, 21], colon cancer [22, 23], pancreatic cancer [24, 25], and cutaneous melanoma [26–29]. In the human liver, normal liver cells barely express the FAP antigen, but it can be highly expressed in aHSCs in livers with fibrosis and cirrhosis. FAP overexpression can enhance the adhesion, metastasis, proliferation, and apoptotic ability of HSCs [30]. Most studies have confirmed that FAP has a tumor-promoting effect and could be a promising target molecule for the diagnosis and treatment of tumors [31]. However, little research has been conducted on the expression and role of FAP in liver tumor nests, and the pathological and physiological significance of FAP in HCC have not been thoroughly studied and remains. Here, we aimed to explore the effect of IL-17a on HSCs and the expression of their functional gene FAP in the tumor microenvironment and to show the cancer-promoting and pro-metastatic mechanism of IL-17a mediated through mesenchymal cells.

## Results

### IL-17a expression is associated with hepatic stellate cells activation in HCC

**Fig. 1:**
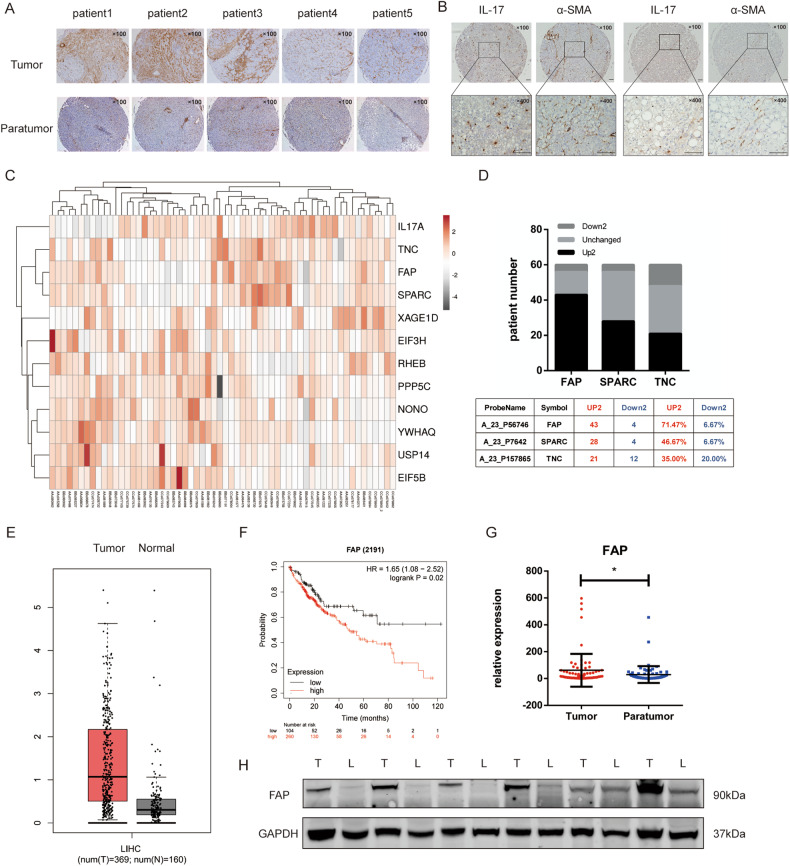
IL-17 is associated with activated hepatic stellate cells. **A** Representative immunohistochemical staining of α-SMA in cancer and adjacent tissues from five HCC patients. Magnification: 100×. **B** Positive correlation between IL-17+ cells and the integrated absorbance of α-SMA (*r* = 0.355, *p* < 0.001), according to immunohistochemical staining. **C** Heatmap of mRNA expression ratio of 12 hepatic stellate cell-related genes in cancer tissues compared to adjacent tissues of 60 HCC patients as determined by on-chip expression profiling. **D** Upregulation of FAP, SPARC, and TNC in patient cancer tissues compared to adjacent tissues. **E** Upregulation of FAP expression in tumor tissues (*N* = 369) compared to normal tissues (*N* = 160) in the LIHC cohort in the TCGA database. **F** Higher FAP expression was associated with worse overall survival in HCC patients. **G** mRNA expression of FAP in cancer and adjacent tissues of 60 HCC patients. **H** Western blot showing FAP expression in cancer and adjacent tissues of 12 HCC patients.

We first used a tissue microarray to detect IL-17+ cells and evaluate the expression of the HSCs activation biomarker actin alpha 2 smooth muscle aorta (ACTA2, also known as α-SMA) in HCC tissues. α-SMA was highly expressed in tumor tissues compared to paratumor tissues (Fig. 1A). Then, correlation analysis of the α-SMA and IL-17 expression levels was conducted based on the IL-17+ cell count and the integrated absorbance of α-SMA. The results showed that there was a correlation between IL-17+ cells and activated stellate cells (*r* = 0.355, *P* < 0.001, Fig. 1B). Since IL-17a functions by binding to IL-17 receptors, we further used the public resources Gene Expression Profiling Interactive Analysis (GEPIA) [32] and Tumor IMmune Estimation Resource 2.0 (TIMER 2.0) [33] to evaluate the correlation between Interleukin 17 Receptor A (*IL-17RA*) and *ACTA2* expression in liver tissues. The results showed a positive correlation between the expression levels of *IL-17RA* and *ACTA2* (GEPIA, GTEx cohort, *r* = 0.43, *P* = 2.3e–06, LIHC Tumor cohort, *r* = 0.19, *P* = 0.00018, LIHC Normal cohort, *r* = 0.38, *P* = 0.0059, Supplementary Fig. 1A–C; TIMER, *r* = 0.13, *P* = 1.2e–02, Supplementary Fig. 1D). Next, we conducted on-chip expression profiling using cancer tissues and adjacent tissues from 60 HCC patients. The results revealed significant differences between the tumor and paratumor tissues regarding the expression of FAP, secreted protein acidic and cysteine rich (SPARC), and tenascin C (TNC), which are closely related to HSCs’ function (Fig. 1C). Among these alterations in gene expression, upregulation of FAP was found in more tissue pairs (Fig. 1D). After searching the TCGA database, we found that the expression of FAP was higher in tumor tissues than in normal tissues (Fig. 1E). Moreover, higher FAP expression was associated with a poorer prognosis in the patients (HR = 1.65(95% CI, 1.08–2.52), *P* = 0.02, Fig. 1F). Then, we examined the mRNA and protein expression of FAP in HCC tissues and paired adjacent tissues. These results also showed that FAP was upregulated in tumor tissues at both the transcriptional and translational levels (Fig. 1G, H). In addition, the expression levels of *ACTA2* and *FAP* were positively correlated, as determined with GEPIA (*r* = 0.658, *P* = 2.29e–47, Supplementary Fig. 1E) and TIMER 2.0 (*r* = 0.64, *P* = 4.1e–61, Supplementary Fig. 1F). Moreover, patients with higher FAP/IL-17a levels had a lower overall survival rate (HR_high _= 1.5, *P* = 0.019, Supplementary Fig. 1G). Therefore, we further speculated that there were reciprocal effects between IL-17a and FAP expression, which activated HSCs and further promoted liver tumorigenesis.

### IL-17a promotes cell activation, proliferation, and metastasis and inhibits apoptosis in the LX2 cell line

**Fig. 2:**
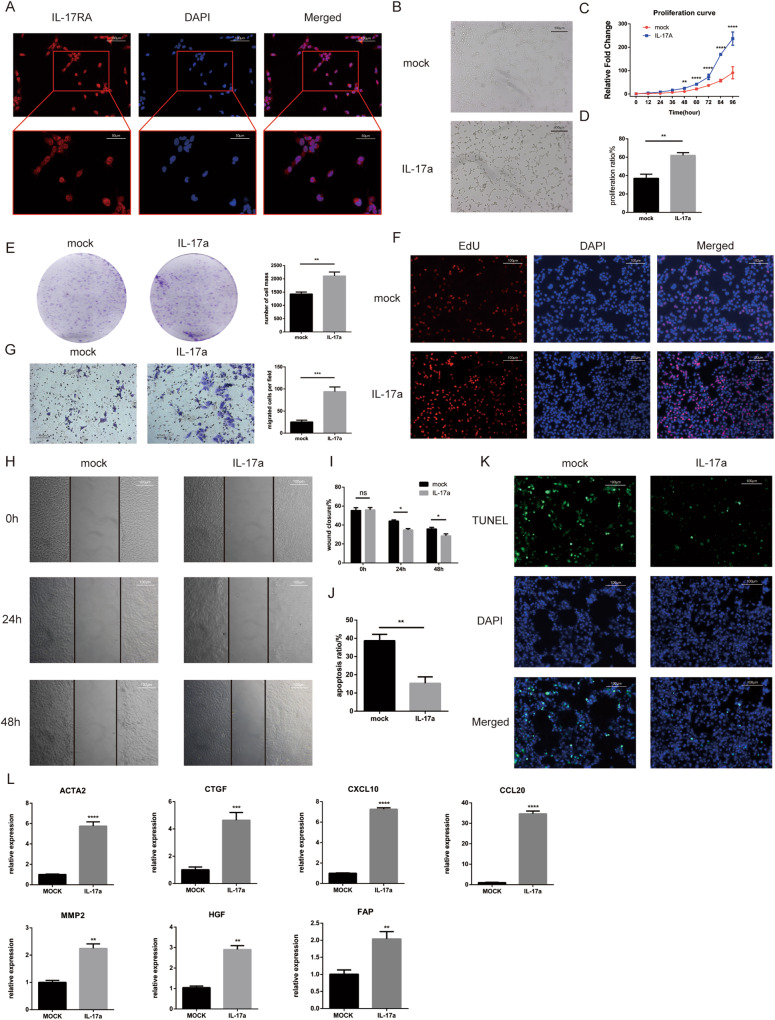
IL-17a activates LX2 cell line, promotes their proliferation and migration, and inhibits their apoptosis. **A** Representative images indicating IL-17RA expression in the LX2 cell line. (Immunofluorescence staining, 200x, blue fluorescence, nuclei; red fluorescence, IL-17RA). **B** Representative images of quiescent (upper) and activated (lower) LX2 cells before and after IL-17a stimulation. **C** Cell proliferation curves of LX2 cells stimulated with 20 ng/ml IL-17a or treated with PBS, as determined by a CCK8 assay. **D** Proliferation rate of cells with IL-17a stimulation or PBS treatment, as determined by an EdU incorporation assay. **E** Representative images and statistical analysis of cell proliferation after IL-17a stimulation or PBS treatment by a plate colony formation assay. **F** Representative images of cell proliferation after IL-17a stimulation or PBS treatment, as shown by EdU incorporation (immunofluorescence staining, 200x, blue fluorescence, nuclei; red fluorescence, proliferating cells). **G** Representative images and statistical analysis of cell migration after IL-17a stimulation or PBS treatment in the Transwell assay. **H** Representative images of cell migration after IL-17a stimulation or PBS treatment in the wound healing assay. **I** Statistical analysis of wound closure after IL-17a stimulation or PBS treatment in the wound healing assay. **J** Apoptosis rate after IL-17a stimulation or PBS treatment, as determined by a TUNEL assay. **K** Representative images of apoptosis in the LX2 cell line after IL-17a stimulation or PBS treatment in the TUNEL assay (immunofluorescence staining, 200x, blue fluorescence, nuclei, green fluorescence, nuclei in cells undergoing apoptosis). **L** mRNA expression of several hepatic stellate cell-related genes in LX2 cell lines after IL-17a stimulation or PBS treatment. *, *p* < 0.05; **, *p* < 0.01; ***, *p* < 0.001.

We first examined the expression of IL-17RA in the LX2 cell line. The immunofluorescence results showed that IL-17RA was highly expressed in the LX2 cell line and was localized in both the cytoplasm and nucleus (Fig. 2A). Then, we treated the LX2 cell line with IL-17a. After treatment, the cell morphology changed, the cell expanded, and the number of protrusions increased (Fig. 2B). The cell colony formation experiment, EdU assay, and CCK8 assays showed that IL-17a promoted the proliferation of LX2 cells (Fig. 2C–F). The wound healing and Transwell assays showed that IL-17a stimulation increased the metastatic ability of LX2 cells (Fig. 2G–I). The TUNEL assay showed that stimulation with IL-17a reduced the apoptosis rate (Fig. 2K, J). The expression of the LX2 activation markers ACTA2 and cellular communication network factor 2 (CCN2, also known as CTGF) was elevated after IL-17a stimulation (Fig. 2L). Likewise, the expression of the inflammatory chemokines C-X-C motif chemokine ligand 10 (CXCL10) and C-C motif chemokine ligand 20 (CCL20) was also increased after IL-17a treatment (Fig. 2L). In addition, the expression levels of hepatocyte growth factor (HGF) and matrix metallopeptidase 2 (MMP2) were increased (Fig. 2L). We also found that the FAP expression was upregulated after IL-17a stimulation (Fig. 2L).

### IL-17a overexpression promotes cell activation, proliferation, and metastasis and inhibits apoptosis in the LX2 cell line

**Fig. 3:**
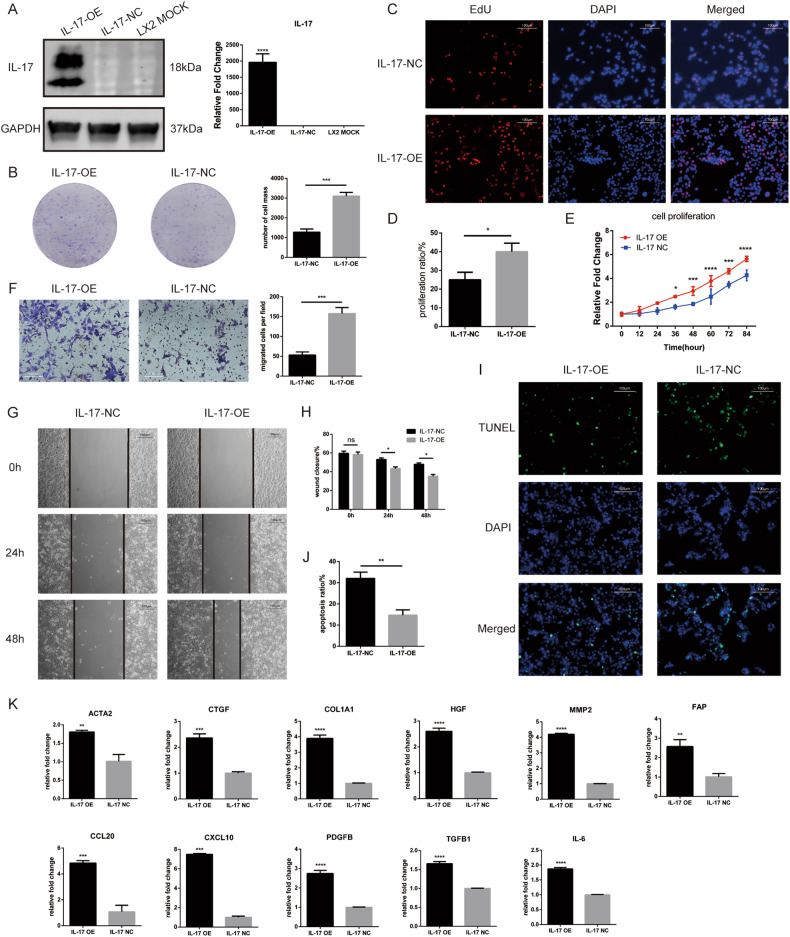
Overexpression of IL-17 activates LX2 cell line, promotes their proliferation and migration, and inhibits their apoptosis. **A** mRNA expression and protein expression levels of IL-17 in the IL-17-overexpressing LX2 cell line (IL-17-OE), the corresponding control cell line (IL-17-NC), and the untreated control cell line (LX2 MOCK). **B** Representative images and statistical analysis of the proliferation of IL-17-OE and IL-17-NC cells by a plate colony formation assay. **C** Representative images of the proliferation of IL-17-OE and IL-17-NC cells in the EdU incorporation assay (immunofluorescence staining, 200x, blue fluorescence, nuclei; red fluorescence, proliferating cells). **D** Proliferation rate of IL-17-OE and IL-17-NC cells in the EdU incorporation assay. **E** Proliferation curves of IL-17-OE and IL-17-NC LX2 cells as determined by a CCK8 assay. **F** Representative images and statistical analysis of the migration of IL-17-OE and IL-17-NC cells in the Transwell assay. **G** Representative images of the migration of IL-17-OE and IL-17-NC cells in the wound healing assay. **H** Statistical analysis of wound closure by IL-17-OE and IL-17-NC cells in the wound healing assay. **I** Representative images of apoptosis in IL-17-OE and IL-17-NC cells in the TUNEL assay (immunofluorescence staining, 200×, blue fluorescence, nuclei; green fluorescence, nuclei in cells undergoing apoptosis). **J** Apoptosis rates of IL-17-OE and IL-17-NC cells in the TUNEL assay. **K** mRNA expression of several hepatic stellate cell-related genes in LX2 cells after IL-17 overexpression. *, *p* < 0.05; **, *p* < 0.01; ***, *p* < 0.001.

For further research, we constructed a cell line with stable overexpression of IL-17a (IL-17-OE) and the corresponding negative control cell line (IL-17-NC) using lentiviruses and verified the overexpression efficiency by qPCR and western blotting (Fig. 3A). The CCK8, colony formation experiment, and EdU assays showed that overexpression of IL-17a promoted the proliferation of LX2 cells (Fig. 3B–E), while the wound healing and Transwell assays confirmed that cell migration was increased (Fig. 3F–H). In addition, the TUNEL assay showed that IL-17a overexpression inhibited apoptosis in the LX2 cell line (Fig. 3I, J). Analysis of the hepatic stellate cell activation markers ACTA2, COL1A1, and CTGF showed that IL-17a overexpression activated LX2 cells, thus elevating the expression of these markers (Fig. 3K). The expression of the inflammatory chemokines CXCL10, CCL20, PDGFB, and IL-6 was also increased after IL-17a overexpression (Fig. 3K). In addition, the expression of LX2 signaling pathway-associated molecules, such as TGFB1, MMP2, and HGF, was elevated after IL-17a overexpression (Fig. 3K). Notably, the expression of FAP was also increased after IL-17a overexpression (Fig. 3K).

### FAP overexpression promotes cell activation, proliferation, and metastasis and inhibits apoptosis in the LX2 cell line

**Fig. 4:**
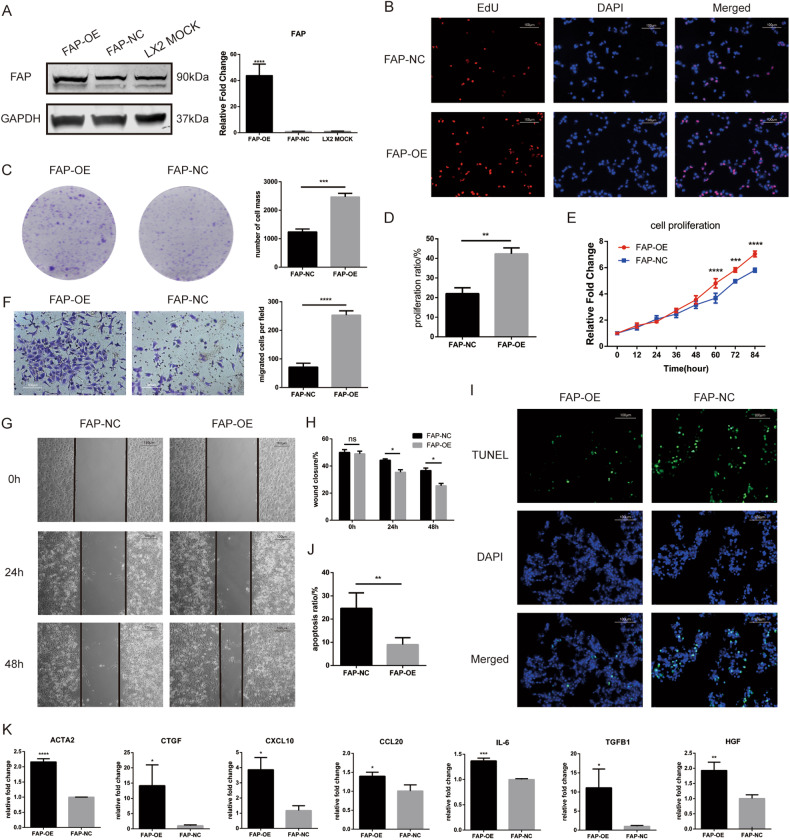
Overexpression of FAP activates LX2 cell line, promotes their proliferation and migration, and inhibits their apoptosis. **A** mRNA expression and protein expression of FAP in the FAP overexpression LX2 cell line (FAP-OE), the corresponding control cell line (FAP-NC) and the untreated control cell line (LX2 MOCK). **B** Representative images and statistical analysis of the proliferation of FAP-OE and FAP-NC cells in the plate colony formation assay. **C** Representative images of the proliferation of FAP-OE and FAP-NC cells in the EdU incorporation assay (immunofluorescence staining, 200x, blue fluorescence, nuclei; red fluorescence, proliferating cells). **D** Proliferation rates of FAP-OE and FAP-NC cells in the EdU incorporation assay. **E** Proliferation curves of FAP-OE and FAP-NC LX2 cells as determined by a CCK8 assay. **F** Representative images and statistical analysis of the migration of FAP-OE and FAP-NC cells in the Transwell assay. **G** Representative images of the migration of FAP-OE and FAP-NC cells in the wound healing assay. **H** Statistical analysis of wound closure by FAP-OE and FAP-NC cells in the wound healing assay. **I** Representative images of apoptosis in FAP-OE and FAP-NC cell lines in the TUNEL assay (immunofluorescence staining, 200x, blue fluorescence, nuclei; green fluorescence, nuclei in cells undergoing apoptosis). **J** Apoptosis rates of FAP-OE and FAP-NC cells in the TUNEL assay. **K** mRNA expression of several hepatic stellate cell-related genes in LX2 cells after FAP overexpression. *, *p* < 0.05; **, *p* < 0.01; ***, *p* < 0.001.

Based on the previous gene expression profile data, we conducted further research on FAP. Considering that the expression of FAP is increased in activated LX2 cells but decreased in quiescent cells, we used lentiviral transduction to construct a FAP overexpression (FAP-OE) cell line and the corresponding control cell line (FAP-NC) and verified the overexpression efficiency by qPCR and western blotting (Fig. 4A). After overexpression of FAP, the colony formation assay, CCK8 proliferation assay and EdU assay showed that FAP promoted the proliferation of LX2 cells (Fig. 4B–E). The wound healing assay and Transwell assay revealed that cell migration was enhanced after FAP overexpression (Fig. 4F–H). The TUNEL assay indicated that apoptosis in the LX2 cell line decreased after FAP overexpression (Fig. 4I, J). Analysis of several markers revealed increases in ACTA2 and CTGF expression, upregulation of the chemokines CXCL10 and CCL20, and increases in the expression of IL-6 and TGFB1 as well as the associated molecule HGF (Fig. 4K).

### IL-17-OE and FAP-OE LX2 cells promoted HCC cell proliferation and migration in vitro

**Fig. 5:**
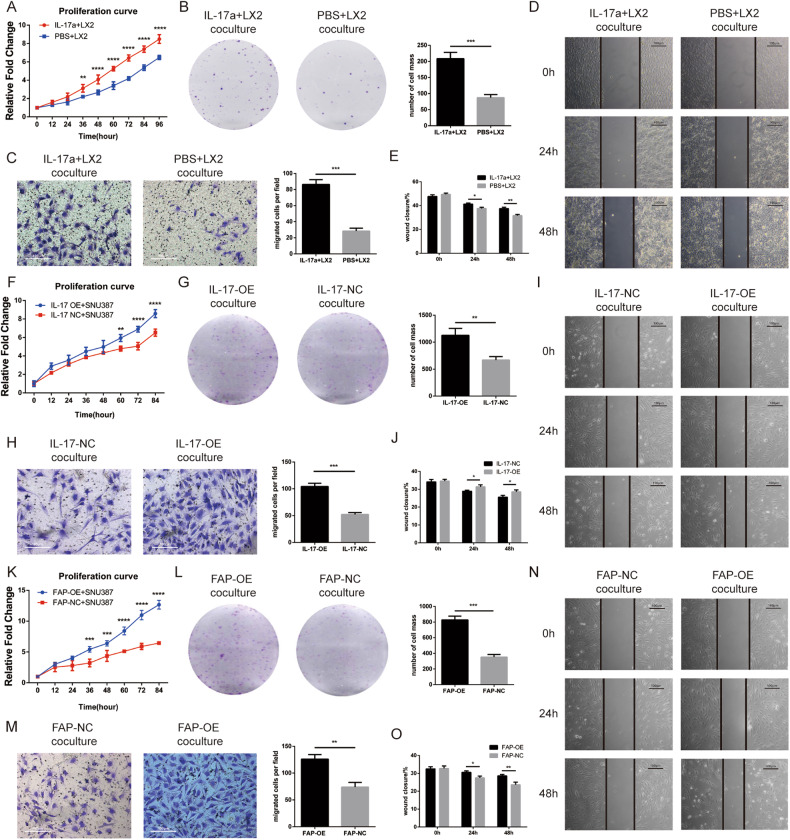
IL-17a stimulation and IL-17 and FAP overexpression in LX2 cells promotes the proliferation and migration and inhibits the apoptosis of SNU387 cells after coculture. **A** Proliferation curve of SNU387 cells after cocultured with IL-17a-stimulated or PBS-treated LX2 cells as determined by CCK8 assay. **B** Representative images and statistical analysis of the proliferation of SNU387 cells after cocultured with IL-17a-stimulated or PBS-treated LX2 cells in the plate colony formation assay. **C** Representative images and statistical analysis of the migration of SNU387 cells after cocultured with IL-17a-stimulated or PBS-treated LX2 cells in the Transwell assay. **D** Representative images of the migration of SNU387 cells after cocultured with IL-17a-stimulated or PBS-treated LX2 cells in the wound healing assay. **E** Statistical analysis of wound closure by SNU387 cells after cocultured with IL-17a-stimulated or PBS-treated LX2 cells in the wound healing assay. **F** Proliferation curve of SNU387 cells after cocultured with IL-17-OE or IL-17-NC LX2 cells as determined by a CCK8 assay. **G** Representative images and statistical analysis of the proliferation of SNU387 cells after cocultured with IL-17-OE or IL-17-NC LX2 cells in the plate colony formation assay. **H** Representative images and statistical analysis of the migration of SNU387 cells after cocultured with IL-17-OE or IL-17-NC LX2 cells in the Transwell assay. **I** Representative images of the migration of SNU387 cells after cocultured with IL-17-OE or IL-17-NC LX2 cells in the wound healing assay. **J** Statistical analysis of wound closure by SNU387 cells after cocultured with IL-17-OE or IL-17-NC LX2 cells in the wound healing assay. **K** Proliferation curve of SNU387 cells after cocultured with FAP-OE or FAP-NC LX2 cells as determined by a CCK8 assay. **L** Representative images and statistical analysis of the proliferation of SNU387 cells after cocultured with FAP-OE or FAP-NC LX2 cells in the plate colony formation assay. **M** Representative images and statistical analysis of the migration of SNU387 cells after cocultured with FAP-OE or FAP-NC LX2 cells in the Transwell assay. **N** Representative images of the migration of SNU387 cells after cocultured with FAP-OE or FAP-NC LX2 cells in the wound healing assay. **O** Statistical analysis of wound closure by SNU387 cells after cocultured with FAP-OE or FAP-NC LX2 cells in the wound healing assay.

Initially, we cocultured the LX2 cell line with the HCC cell line SNU387. The LX2 cells were added into the upper chamber of the 6-well plate, and the SNU387 cells was put into the lower well of the 6-well plate. The CCK8 and colony formation assays showed that stimulation of LX2 cells with IL-17a increased their proliferation ability (Fig. 5A, B). The Transwell assay showed that under these coculture conditions, SNU387 cells treated with IL-17a migrated more readily (Fig. 5C), which was similarly proven by the wound healing assay (Fig. 5D, E). Then, we evaluated the proliferation and migration abilities of SNU387 cells cocultured with IL-17-OE cells. Coculture with IL-17-OE cells increased the proliferation and migration abilities of HCC cells, similar to the effects of IL-17a stimulation (Fig. 5F–J). In addition, the CCK8 and colony formation assays showed that FAP overexpression increased the proliferation ability of tumor cells (Fig. 5K, L), while the Transwell and wound healing assays suggested that FAP upregulation promoted the migration of tumor cells (Fig. 5N–O).

### IL-17-OE and FAP-OE- LX2 cells promoted the development and progression of liver tumors in vivo

**Fig. 6:**
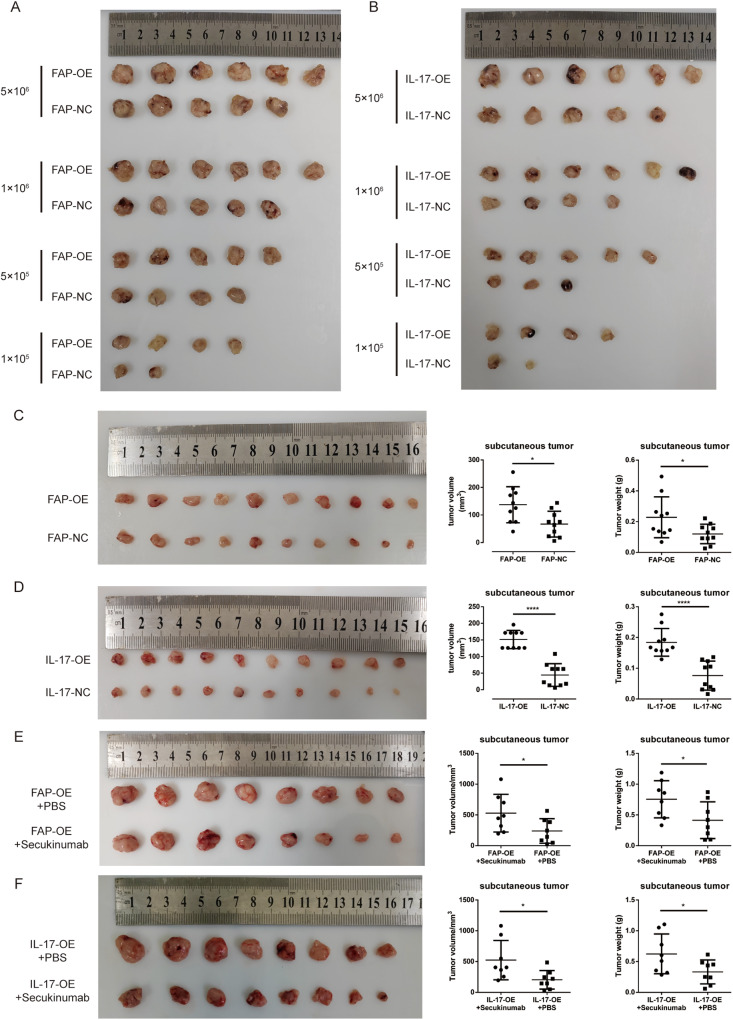
IL-17a stimulation and IL-17 or FAP overexpression in LX2 cells promote liver tumor formation and progression in vivo. **A** Number and size of subcutaneous tumors in nude mice implanted with varying numbers of SNU387 cells cocultured with IL-17-OE or IL-17-NC cells. **B** Number and size of subcutaneous tumors in nude mice implanted with varying numbers of SNU387 cells cocultured with FAP-OE or FAP-NC cells. **C** The tumor size, tumor weight, and their corresponding statistical analysis of nude mice implanted with equal numbers of SNU387 cells cocultured with FAP-OE or FAP-NC cells. **D** The tumor size, tumor weight, and their corresponding statistical analysis of nude mice implanted with equal numbers of SNU387 cells cocultured with IL-17-OE or IL-17-NC cells. **E** The tumor size, tumor weight, and their corresponding statistical analysis of nude mice implanted with equal numbers of SNU387 cells cocultured with FAP-OE cells after treated with Secukinumab or PBS. **F** The tumor size, tumor weight, and their corresponding statistical analysis of nude mice implanted with equal numbers of SNU387 cells cocultured with IL-17-OE cells after treated with Secukinumab or PBS.

Then, in order to verify the ability of these alterations in LX2 cells to promote HCC development and progression, we used the IL-17a and FAP overexpression cell lines (IL-17-OE and FAP-OE) and their negative control cell lines (IL-17-NC and FAP-NC) with the HCC cell line SNU387 at an LX2 cell: SNU387 cell ratio of 1:5 to carry out the subcutaneous tumorigenesis assay in mice. The extreme limiting dilution assay performed in vivo showed that FAP overexpression promoted liver tumor formation (Fig. 6A), with the tumor initiation frequency of these cells found to be much higher than that of the corresponding negative control cells (Supplementary Table 1). Similarly, overexpression of IL-17a facilitated liver tumor formation (Fig. 6B), and the tumor initiation frequency of the IL-17-OE cells was much higher than that of the corresponding control cells (Supplementary Table 2). Moreover, we used the same number of SNU387 and LX2 cells in experimental and control groups to establish subcutaneous tumor models, and the results showed that the volume of tumors formed from FAP-OE and IL-17-OE cells was much larger than that of tumors formed from the corresponding negative control cells. Apart from that, the tumor weight was much higher in FAP-OE and IL-17-OE groups than the corresponding control groups (Fig. 6C, D). In addition, we intratumorally injected the Secukinumab (3 mg/kg) or PBS to validate the IL-17a’s cancer-promoting ability. The Secukinumab is a kind of human monoclonal antibody that selectively binds to and neutralizes IL-17a in vivo. The results showed that, after the Secukinumab administration, the volume of the subcutaneous tumor was smaller and the weight of the tumor was lighter in either FAP-OE or IL-17-OE models (Fig. 6E, F).

### IL-17 promotes FAP expression by activating the p-STAT3 signaling pathway

**Fig. 7:**
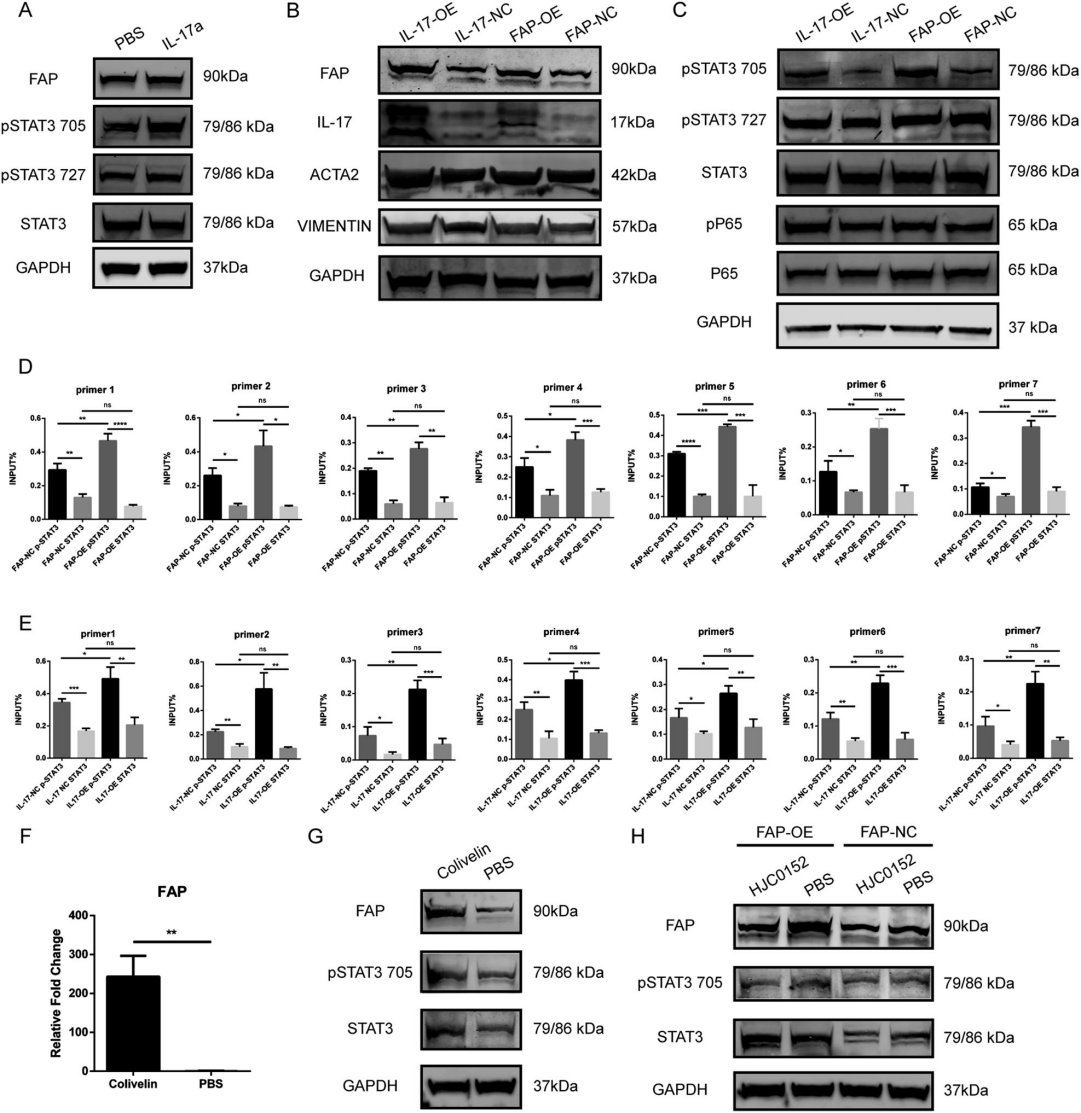
IL-17 upregulates FAP expression by activating the p-STAT3 signaling pathway. **A** Protein levels of FAP, pSTAT3 (Tyr705), pSTAT3 (Ser727), and STAT3 after 20 ng/ml IL-17a stimulation or PBS treatment. **B** Protein levels of FAP, IL-17, ACTA2, and VIMENTIN in IL-17-OE cells, FAP-OE cells, and the corresponding negative control cell lines. **C** Protein levels of pSTAT3 (Tyr705), pSTAT3 (Ser727), STAT3, pP65, and P65 in IL-17-OE cells, FAP-OE cells, and the corresponding negative control cell lines. **D** The binding ability of pSTAT3 (Tyr705) and STAT3 to the FAP promoter region in FAP-OE and FAP-NC cells was evaluated by a CUT&RUN assay. **E** The binding ability of pSTAT3 (Tyr705) and STAT3 to the FAP promoter region in IL-17-OE and IL-17-NC cells was evaluated by a CUT&RUN assay. **F** The mRNA expression of FAP of LX2 cell line after 24 h treatment of 50 μg/mL Colivelin. **G** The expression of FAP, STAT3, and p-STAT3 in LX2 cell line after 24 h treatment of 50 μg/mL Colivelin. **H** The FAP, STAT3, p-STAT3 level in FAP-OE and FAP-NC cell lines after 2 μM HJC0152 treatment.

As mentioned above, FAP expression was increased by IL-17a stimulation or IL-17a overexpression, and we reexamined this upregulation using western blotting (Fig. 7A, B). Next, we tried to determine the mechanism of this upregulation. Previous studies found that IL-17a acts on liver tumor cells through the STAT3 signaling pathway, further interacting with the NF-κB signaling pathway. We thus suspected whether the same regulatory mechanism exists in HSCs. To this end, we measured the levels of related proteins in the STAT3 and NF-KB signaling pathways in different cell lines. After IL-17a stimulation, the level of pSTAT3-Tyr705 was increased, while the levels of pSTAT3-Ser727 and STAT3 remained unchanged (Fig. 7A). The levels of pSTAT3-Tyr705 and p-p65 were elevated and those of pSTAT3-Ser727, total STAT3, and p65 remained stable in the IL-17-OE and FAP-OE cell lines compared to the IL-17-NC and FAP-NC-cell lines, indicating activation of the STAT3 and NF-KB signaling pathways (Fig. 7C). Moreover, we found that the expression of ACTA2 was increased after overexpression of either IL-17a or FAP (Fig. 7B). Furthermore, we searched a transcription factor prediction website and found that NFκB and STAT3 might act as transcription factors of FAP and regulate its expression (Supplementary Fig. 2). Next, we used the JASPAR database to predict the binding sites between the STAT3 transcription factor and the FAP promoter region (Supplementary Table 3). Based on the binding site sequence, we designed upstream and downstream primers for a cleavage under targets and release using nuclease (CUT&RUN) assay. The results of this assay showed that STAT3 could bind to the promoter region of FAP and that the amount of bound activated p-STAT3 was higher than that of nonphosphorylated STAT3 (Fig. 7D, E). Similarly, we used IL-17-OE cells and the corresponding control cell line to quantify the binding of STAT3 proteins to the FAP promoter region. Compared with the control cell line, the IL-17-OE cell line exhibited increased binding of p-STAT3 to the FAP promoter after IL-17a overexpression (Fig. 7D). Furthermore, we quantified the binding of STAT3 and FAP in the FAP-OE LX2 cell line and the corresponding control cell line. The binding ability of p-STAT3 was also greater in FAP-OE cells (Fig. 7E). These results suggested that after IL-17a stimulation, the STAT3 signaling pathway was activated, and the phosphorylation level of STAT3 increased. The binding of the transcription factor p-STAT3 to the FAP promoter region was increased, thus increasing the transcription of FAP and promoting FAP protein expression. In order to further validate the activation role of STAT3 to FAP expression, we used a STAT3 signaling pathway activator, Colivelin, to treat the LX2 cell line to testify the expression of FAP and STAT3. The results showed that, after 24 h treatment of 50 μg/mL Colivelin, the mRNA expression of FAP was upregulated significantly (Fig. 7F). Then we examined the activation status of STAT3 signaling pathway and the expression of FAP protein. Not surprisingly, the p-STAT3 level was potently upregulated and the total STAT3 level upregulated slightly. In addition, the FAP protein level was also raised after the Colivelin administration (Fig. 7G). Likely, we used HJC0152, a STAT3 inhibitor, to treat FAP-OE cells and the corresponding negative control cell line to further testify the regulation role of STAT3 to FAP expression. After 2 μM HJC0152 treatment, the p-STAT3 level was decreased significantly and the STAT3 signaling pathway was effectively inhibited, thus leading to a downregulation of FAP expression in both cell lines (Fig. 7H). The experiments above demonstrated that STAT3 was a potent activator of FAP expression.

## Discussion

Many studies on the relationship between IL-17 and HSCs have been conducted in the context of hepatitis and liver cirrhosis [34–36]. IL-17 can accelerate the hepatitis-liver fibrosis-cirrhosis axis by activating HSCs [37]. Regarding the immune microenvironment of liver cancer, few studies involving IL-17 cells, HSCs, and their functional genes have been conducted. IL-17+ cells, like HSCs, are also stromal cells in the tumor microenvironment. Most IL-17+ cells are Th17 cells, which are CD4^+^ effector T cells. The functions of Th17 cells and IL-17+ cells have attracted much attention in recent years [13, 38–40]. Their roles and mechanisms in various immune pathologies and resistance to specific microbial infections have been gradually clarified by many studies. Since Th17 cells and IL-17+ cells are important participants in the inflammatory tumor immune microenvironment, their roles in tumor occurrence and development have been hot topics in recent research [41]. In the context of liver cancer, our previous studies have shown that Th17 cells and IL-17+ cells can significantly promote the development and progression of liver cancer [14, 42, 43]. To date, many studies have focused on the direct interaction between IL-17a and tumor cells, but there is no clear evidence showing that IL-17a can promote the growth, invasion, and metastasis of liver cancer cells by acting on HSCs. Thus, we studied the effect of IL-17a on liver nonparenchymal cells by using the cytokine IL-17a to stimulate LX2 cells or overexpressing IL-17a in LX2 cells. Both IL-17a stimulation and IL-17a overexpression activated LX2 cells, leading to changes in their proliferation, apoptosis, and metastasis capabilities. Moreover, our in vitro and in vivo experiments showed that IL-17-OE cells and IL-17a-stimulated LX2 cells cocultured with HCC cells promoted the occurrence and progression of HCC cells.

In addition, the on-chip expression profiling showed that the expression of the hepatic stellate cell-related genes FAP, SPARC, and TNC, especially FAP, was upregulated in tumor tissues compared to normal tissues. This change in FAP expression led to alterations in the activation status of HSCs. We found that FAP induced the activation of HSCs and that overexpression of FAP promoted the proliferation and migration and inhibited the apoptosis of HSCs. In vivo and in vitro experiments have shown that upregulation of FAP can promote the occurrence and development of tumors. In addition, studies have shown that FAP can degrade dipeptides and type I collagen in the extracellular matrix [44]. FAP also has nonenzymatic catalytic activity and exerts effects such as promoting the proliferation of tumor cells, degrading and reconstructing the extracellular matrix, establishing the tumor vasculature, and mediating the immunosuppressive tumor environment [45]. Previous experiments on pancreatic cancer have shown that targeting FAP could not only improve the body’s antitumor immune function but also enhance the efficacy of antitumor drugs [25].

Previous studies have shown that IL-17 contributes to the occurrence of tumors by affecting the activation status of the STAT3 signaling pathway in tumor cells [46, 47]. However, the effect of IL-17a on liver stromal cells has not been clearly reported. In our research, we found that the expression of FAP was increased after IL-17a stimulation or IL-17a overexpression and that the level of pSTAT3 was also increased. As a classical transcription factor, STAT3 binds to the FAP promoter region and promotes the transcription of FAP. Therefore, we assumed that IL-17a stimulation leads to the upregulation of FAP expression, activation of stellate cells, and promotion of the growth and proliferation of HCC cells in the tumor microenvironment.

Our research has many limitations. First, Th17 cells are a subset of CD4 + T cells discovered through their secretion of IL-17, but all IL-17-secreting cells are not Th17 cells. Numerous studies have found that Tc17 cells (CD3+, CD4-, CD8+, IL-17a+) [48], γδT cells (CD3+, CD4-, CD8-, γδTCR+, αβTCR-, IL-17a+) [49, 50], neutrophils (CD15+/Gr-1hi) [51, 52], mast cells (CD163+ or MCT+) [53, 54], innate lymphocytes (CD127+) [51] and many other cells can also secrete IL-17a. We did not distinguish these cells separately but explored their relationship with liver fibrosis and liver cancer as a whole population. Future studies should distinguish these cells by flow cytometry or immunohistochemistry and use human or mouse primary cells to study the relationship between a specific kind of IL-17+ cell and liver fibrosis. In addition, the results of our in vivo experiments on the promoting roles of FAP overexpression and IL-17a upregulation should be further validated in genetically engineered mice with high IL-17a or FAP expression in HSCs using a chemically induced liver cancer model to make the results more convincing. Moreover, an anti-IL-17 mAb, secukinumab, can be used to restore the cancer-promoting effects of IL-17a and treat psoriasis in clinical practice [55, 56]. Previous studies have also found that secukinumab has inhibitory effects on the growth and metastasis of liver cancer in mice [42]. Therefore, in future studies, HSCs should be treated with this antibody, and its effect on the incidence of subcutaneous liver tumor formation should be evaluated. Finally, the specific mechanism underlying the effect of FAP overexpression in HSCs on the development and progression of liver tumors has not been fully explained. The effect of FAP overexpression on alterations in substances and molecules in the tumor microenvironment needs to be further confirmed. The relationship among IL-17a, STAT3, and FAP should also be studied in greater depth.

## Materials and methods

### Subcutaneous tumorigenesis mouse model

Four- to six-week-old nude male mice (BALB/c nude) were purchased from GemPharmatech^TM^ Company (Nanjing, China). Forty-eight nude mice were randomly divided into 4 groups, with 12 mice in each group. The nude mice in each group were injected in the sub-axillary region of the right forelimb with a mixture of tumor cells (SNU387) and HSCs (FAP-OE cells, the corresponding control cells (FAP-NC), IL-17-OE cells or the corresponding negative control cells (IL-17-NC)) at a 5:1 ratio, with a total of 5 × 10^6^, 1 × 10^6^, 5 × 10^5^, and 1 × 10^5^ cells/mouse injected in the FAP-OE, FAP-NC, IL-17-OE and IL17-NC groups, respectively. Another 40 nude mice were injected with a mixture of 5 × 10^5^ SNU387 tumor cells and HSCs at a 5:1 ratio with Matrigel for subcutaneous tumorigenesis. Apart from that, a total of 32 nude mice were randomly divided into four groups, with 8 mice in each group to intratumorally receive either 100 μL Secukinumab (3 mg/kg) or PBS. These four groups of mice were injected with a mixture of 5 × 10^6^ SNU387 tumor cells and IL-17-OE or FAP-OE cells at a 5:1 ratio. Subcutaneous tumors were harvested from the mice 2 months after cell injection. The sizes and weight of the tumors in each group were recorded after the mice were sacrificed. All animal experiments were approved by the Animal Care Committee of Naval Medical University, and the investigation complied with the US National Institutes of Health Guide for the Care and Use of Laboratory Animals.

### Human liver tissue

A total of 150 specimens collected from 75 patients with HCC were selected from the liver tissue specimen bank of Eastern Hepatobiliary Surgery Hospital. 60 patients’ samples were used to perform gene expressing profile. HCC tumor tissues (taken from the site of robust tumor growth) and matched adjacent tissues (taken from a site more than 2 cm from the tumor margin) were collected and used for on-chip expression profiling. All patients who provided liver samples signed an informed consent form before surgery and were reported to the hospital ethics committee for approval.

### Cell culture and experimental conditions

The human HCC cell line Huh7 and the human stellate cell line LX2 were purchased from the Chinese Academy of Sciences Cell Bank. These cells were cultured in high-glucose Dulbecco’s modified Eagle’s medium containing 10% fetal bovine serum (FBS) at 37 °C in an atmosphere containing 5% CO_2_.

### The coculture system of LX2 cells and HCC cells

The stimulation of IL-17a to the LX2 cell lines was performed before the coculture operation. The coculture system was established using 6-well culture plate with a culture chamber in each well. The upper chamber separated two kinds cells with a PC membrane, where the pore diameter was 0.4 μm, allowing cells to contact to each other and permitting small molecules to move across, but not allowing cells to shuttle back and force. The LX2 cells were added into the upper chamber of the 6-well plate, and the SNU387 cells was put into the lower well of the 6-well plate. The two kinds of cells were cocultured using complete culture media for 48 h. Then the SNU387 cells were used for later experiments.

### Human gene expression profiling

Human expression profiling was performed using an Agilent Whole Human Genome Oligo Microarray (4 × 44 K) (Agilent, USA) by Shanghai Biotechnology Corporation (Shanghai, China). The microarray was used to determine the expression profiles of over 41,000 human genes and transcripts in tumor and paratumor tissues of 60 patients. In brief, total RNA was extracted and purified before amplification and labeling. Each slide was then hybridized and scanned with an Agilent Microarray Scanner (Agilent Technologies, US) with default settings, Dye channel: Green, Scan resolution: 5 μm, PMT: 100% and 10%, 16 bit. Raw data were normalized with the quantile algorithm in GeneSpring 11.0 software (Agilent Technologies, US, RRID:SCR_009196).

### Lentiviral transduction

A stable FAP-overexpressing cell line (FAP-OE) and the corresponding control cell line (FAP-NC) and a stable IL-17a-overexpressing cell line (IL-17-OE) and the corresponding control cell line (IL-17-NC) were constructed using the hepatic stellate cell line LX2. The FAP overexpression lentivirus was synthesized and packaged by OBiO Technology (Shanghai, China). The overexpression vector was constructed in the pcSLenti-EF1-EGFP-P2A-Puro-CMV-MCS-3×FLAG-WPRE plasmid, and the virus titer was 2.56E + 8 TU/ml. The IL-17a overexpression lentivirus was synthesized and packaged by Genomeditech (Shanghai, China). The overexpression vector was constructed in the PGMLV-CMV-MCS-EF1-ZsGreen1-T2A-Puro plasmid, and the virus titer was 1.0E + 8 TU/ml. Transfection was conducted when the cells were 60–70% confluent. The virus was added to the cell plate, and polybrene (5 μg/ml) was added to promote infection. Fluorescence microscopy was used to determine the infection efficiency after 72 h, and stably transduced cells were selected with puromycin.

### Cleavage under targets & release using nuclease (CUT&RUN)

A CUT&RUN Assay Kit (CST, #86652) was used according to the user manual. The antibodies used were as follows: anti-STAT3 (Cell Signaling Technology Cat# 4904, RRID: AB_331269) and anti-phospho-STAT3 (Tyr705) (D3A7) (Cell Signaling Technology Cat# 9145, RRID: AB_2491009). The qPCR primers used for cleavage under targets & release using nuclease (CUT&RUN) were as follows:

**Table:** 

Primer name	Sequences	
	Forward (5′–3′)	Reverse (5′–3′)
STAT3-FAP-1	CGACATCTTTATTTCTGCAGTCAG	GGTTGGGTGAGTCAAGCTGT
STAT3-FAP-2	ACAGCTTGACTCACCCAACC	AAGGGTGGGAAATGATGAGA
STAT3-FAP-3	CCTGTGTACTCTGGGGCTTT	GATTGTCTTCTCTGGAACACACC
STAT3-FAP-4	CCAGCCACCAGGAATACAGT	CCCTGAATTCCAGCCTTATG
STAT3-FAP-5	AATTCAGGGAGGGATGTCTG	TACCACGGGACACTTTCTCC
STAT3-FAP-6	GCCATTTCCATTCTTCCAAA	TTTAGTATGGGTTAGGTGTGTATGC
STAT3-FAP-7	CAGTTCATTTGAGGGCCAAG	CCACGGACTTTTGAATACCG

### Cellular apoptosis detection (TUNEL assay)

Five thousand cells per well were seeded into 24-well plates. The H2O2 (terminal concentration: 1 mmol/L) were used to induce cell apoptosis. When the cells were 60% confluent, the TUNEL Apoptosis Detection Kit (Beyotime, #C1086) was used to quantify apoptosis. The kit was applied according to the user’s manual. The cells were fixed, stained, and observed under a microscope for imaging.

### Cell proliferation assay (CCK8)

Two thousand cells per well were seeded and maintained in 96-well plates. At the indicated time points (0, 12, 24, 48, 60, 72, 84, and 96 h after adherence), Cell Counting Kit 8 (CCK8; Dojindo Laboratories, #CK04) solution was mixed with culture medium at a 1:10 ratio and added to each well, and the plates were incubated for 1 h. Then, each plate was placed into a microplate reader, and the absorbance at 450 nm was measured.

### Cell proliferation assay (EdU incorporation)

Five thousand cells per well were seeded into 24-well plates. The BeyoClick™ EdU Cell Proliferation Kit with Alexa Fluor 555 (Beyotime, #c0075) was used to determine the cell proliferation status when the cells were 60–70% confluent. The assay kit was used according to the manufacturer’s instructions. The cells were fixed, stained, and observed under a microscope for imaging.

### Western blot analysis

Cells were lysed with RIPA lysis buffer (Beyotime, P0013C) to obtain total protein, and the protein concentration was then quantified by the BCA method. Then, 30 μg of protein from each sample was separated by SDS‒PAGE and transferred to a PVDF membrane for detection. The antibodies used were as follows: anti-FAP1 (Affinity Biosciences Cat# AF5344, RRID: AB_2837829), anti-GAPDH (Affinity Biosciences Cat# T0004, RRID: AB_2833041), anti-IL17a (R&D Systems, #AF-317-NA), anti-Stat3 (79D7) (CST, #4904), anti-phospho-Stat3 (Tyr705) (D3A7) (CST, #9145), anti-phospho-Stat3 (Ser727) (CST, #9134), anti-vimentin (D21H3) (CST, #5741), anti-NF-κB p65 (D14E12) (CST, #8242), anti-phospho-NF-κB p65 (Ser536) (93H1) (CST, #3033), anti-α-smooth muscle actin (D4K9N) (CST, #19245), IRDye800CW goat anti-rabbit IgG (LICOR, #926-3221), and IRDye680LT goat anti-mouse IgG (LICOR, #926-68020).

### Quantitative reverse transcription PCR (qRT–PCR)

Total RNA of cells was extracted with TRIzol (Invitrogen), and cDNA synthesis was performed with the Takara reverse transcription kit (Takara, RR036A). The QuantStudio^TM^ 5 system was used for qRT-PCR analysis. The primers used are as follows:

**Table:** 

Gene	Forward (5′→3′)	Reverse (5′→3′)
FAP	CAAAGGCTGGAGCTAAGAATCC	ACTGCAAACATACTCGTTCATCA
GAPDH	AGCGAGCATCCCCCAAAGTT	GGGCACGAAGGCTCATCATT
TGFB1	GGCCAGATCCTGTCCAAGC	GTGGGTTTCCACCATTAGCAC
IL-6	ACTCACCTCTTCAGAACGAATTG	CCATCTTTGGAAGGTTCAGGTTG
MMP2	GATACCCCTTTGACGGTAAGGA	CCTTCTCCCAAGGTCCATAGC
CXCL10	AGAGTGTCTGCGGATACTTCC	CCAACAGTGTAGGTCTTGGTG
CCL20	TGCTGTACCAAGAGTTTGCTC	CGCACACAGACAACTTTTTCTTT
HGF	GCTATCGGGGTAAAGACCTACA	CGTAGCGTACCTCTGGATTGC
IL-17a	AGATTACTACAACCGATCCACCT	GGGGACAGAGTTCATGTGGTA
ACTA2	CTATGAGGGCTATGCCTTGCC	GCTCAGCAGTAGTAACGAAGGA
PDGFB	CTCGATCCGCTCCTTTGATGA	CGTTGGTGCGGTCTATGAG
CTGF	CAGCATGGACGTTCGTCTG	AACCACGGTTTGGTCCTTGG
COL1A1	GAGGGCCAAGACGAAGACATC	CAGATCACGTCATCGCACAAC

### Plate colony formation assay

Cells were seeded (2000 cells/well) and maintained in a 6-well plate with FBS-free medium for 2 weeks. The cells were then fixed with formaldehyde and stained with crystal violet solution for 15 min. The cell clusters in each well were counted and photographed.

### Immunofluorescence staining

Cells were seeded on cover slips in 6-well plates. When the cells were ~60% confluent, they were sequentially stained with a primary antibody (Affinity, #DF3602), secondary antibody (LICOR, #926-3221), and DAPI according to the user manuals. A confocal laser microscope was used for imaging.

### Tissue microarrays (TMAs) and immunohistochemistry

To examine the relationship between the density of intratumoral IL-17+ cells and α-SMA expression, 286 cases were chosen for immunohistochemical staining using serial whole tumor sections. TMAs were constructed as described elsewhere using tissues from the abovementioned cases. Immunohistochemical staining was conducted with the following primary monoclonal antibodies (mAbs): goat anti-human IL-17a (R&D Systems, #AF-317-NA) and rabbit anti-human α-Smooth Muscle Actin (Cell Signaling Technology). Blank controls were treated identically, except the primary antibodies were omitted.

IL-17 and α-SMA staining in TMAs was evaluated at 200× magnification using light microscopy by two investigators blinded to the clinicopathologic data of the patients. For IL-17 immunostaining, the number of positively stained cells in each 1-mm diameter cylinder was calculated manually and expressed as the mean value of duplicates (cells/1-mm core). To calculate the intensity of α-SMA staining, the integrated absorbance and the area of positive staining in each 1-mm diameter cylinder were measured using Image-Pro Plus v6.0 software (Image-Pro Plus, RRID:SCR_007369). The mean α-SMA density was calculated as the product of the integrated absorbance and the total area.

### Cell migration assay (Transwell assay)

Ten thousand cells were resuspended in 200 µl of FBS-free medium and seeded in the upper chambers of a 24-well plate. Then, 500 µl of complete medium was added to the lower chambers. The chambers were collected after 48 h, and the cells attached to the chamber membrane were fixed and stained with crystal violet solution for 15 min. The cells were counted and imaged using a microscope.

### Cell migration assay (wound healing assay)

2×10^6^ cells were added to one well of a 6-well plate, and after the cells are completely adherent, the pipette tip were used to draw a straight line in the center of the well. The suspended cells were washed off with PBS. The serum-free medium was added into the well to keep cells alive. The pictures were shot at 24 h and 48 h using a microscope with camera.

### Statistical analysis

All the data are expressed as the mean ± SD of at least three independent experiments and were analyzed by two-tailed Student’s *t* test, one-way ANOVA or two-way ANOVA. A nonparametric test was used when the data did not meet the normal distribution and homogeneity of variance assumptions. All statistical analyses were performed using GraphPad Prism 7.0 and relevant R packages. Probability (P) values of ≤0.05 were considered to indicate statistical significance. *, *p* < 0.05; **, *p* < 0.01; ***, *p* < 0.001; ****, *p* < 0.0001.

### Supplementary information

Western blot image

Supplementary Figure 1

Supplementary Figure 2

Supplementary information

## Data Availability

The datasets used and analyzed during the current study are available from the corresponding author upon reasonable request.
