# Supplementary figures and images for: IL-17a promotes hepatocellular carcinoma by increasing FAP expression in hepatic stellate cells via activation of the STAT3 signaling pathway
Source: Cell Death Discov. 2024 May 13;10:230. doi: 10.1038/s41420-024-01995-4 (PMC11091202; doi:10.1038/s41420-024-01995-4)

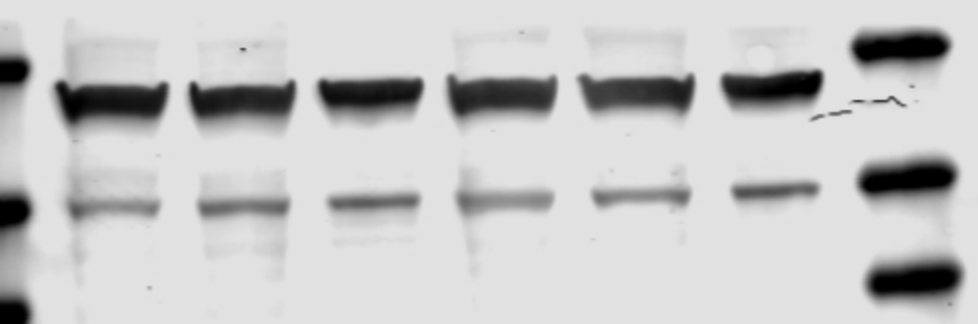

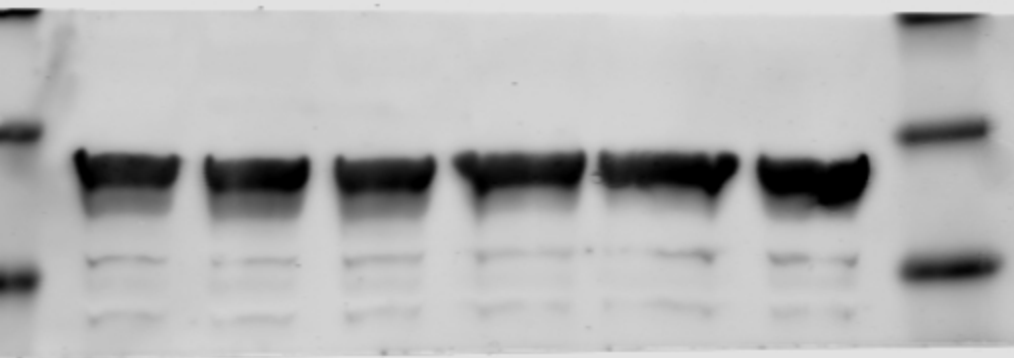

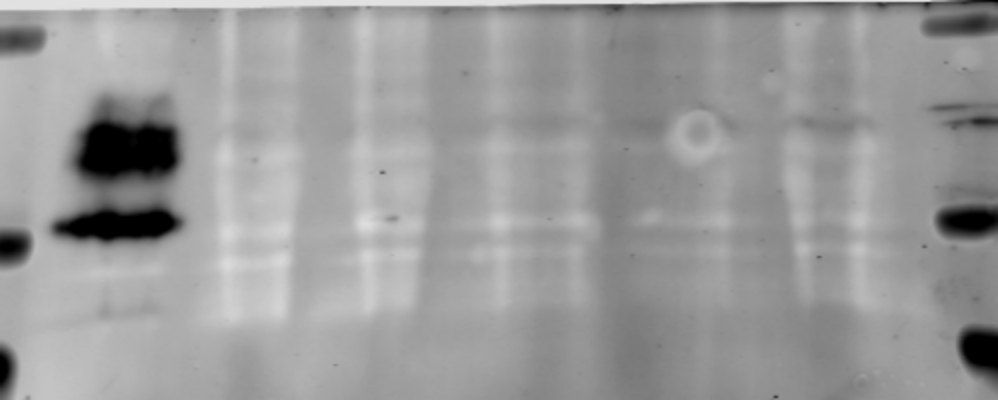

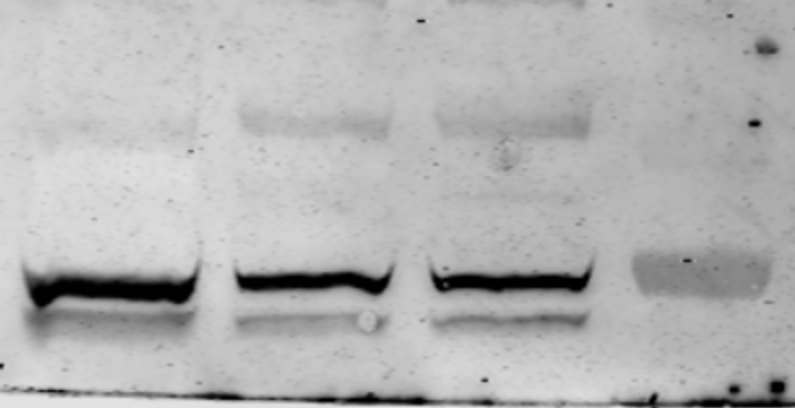

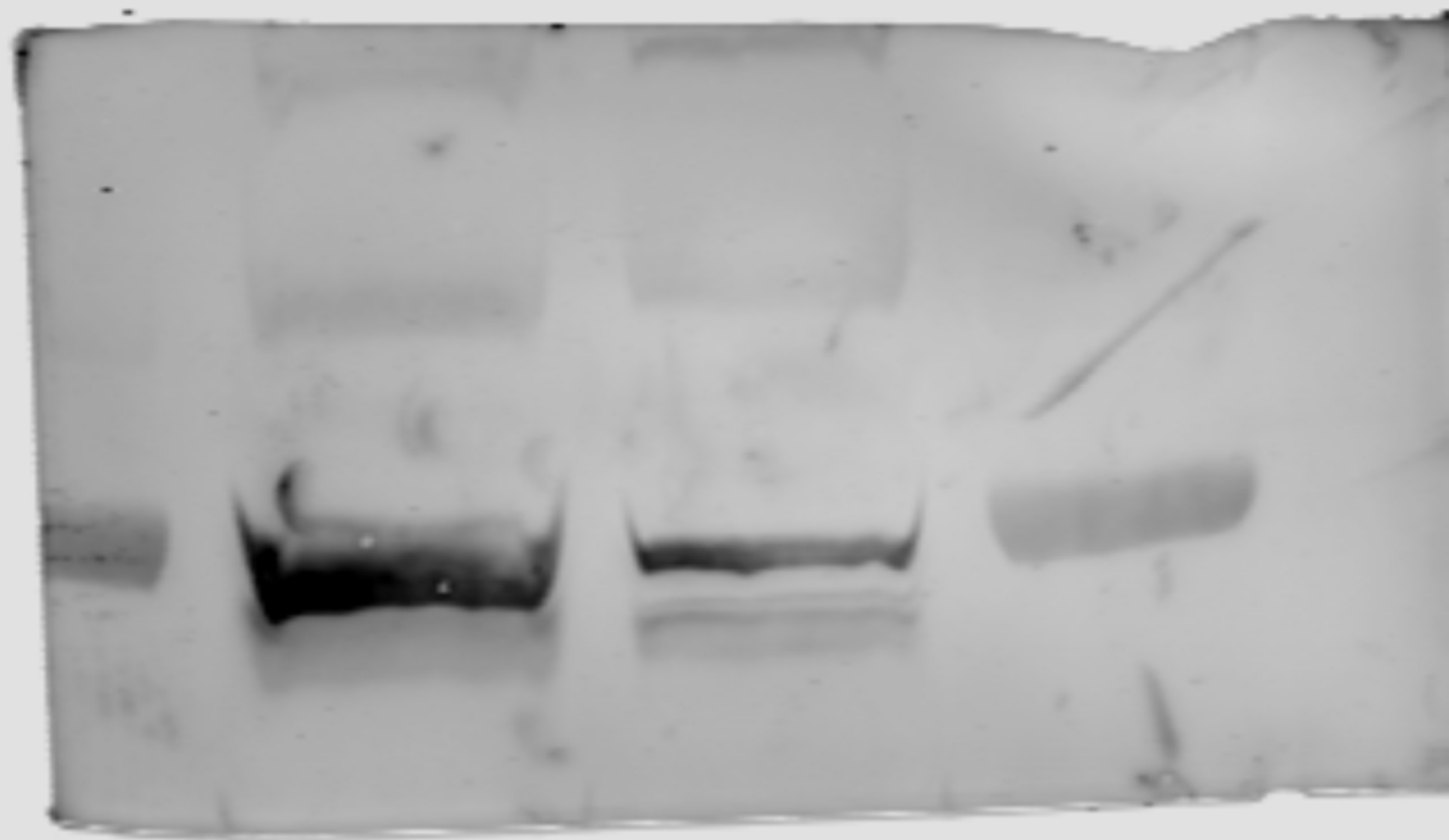

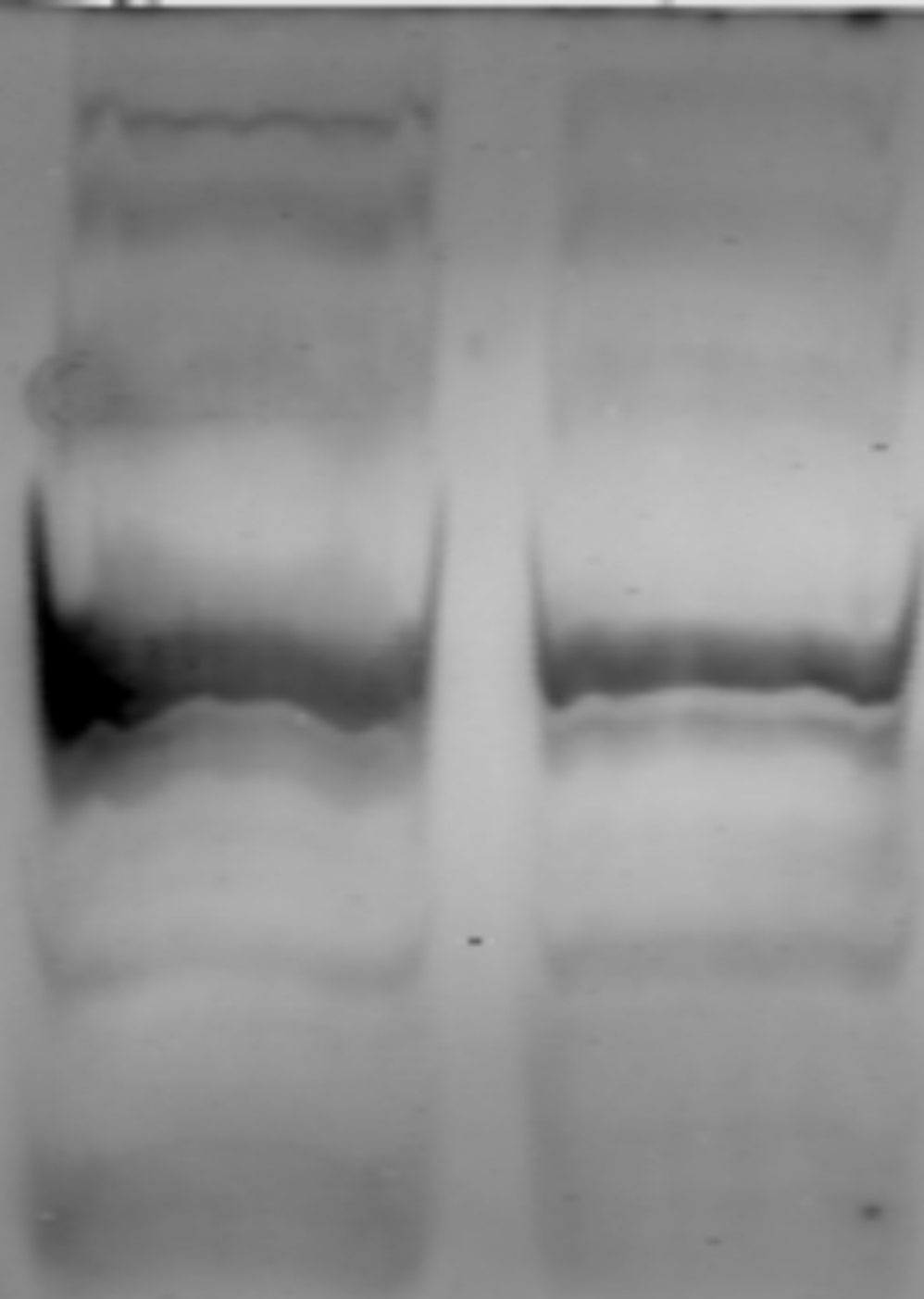

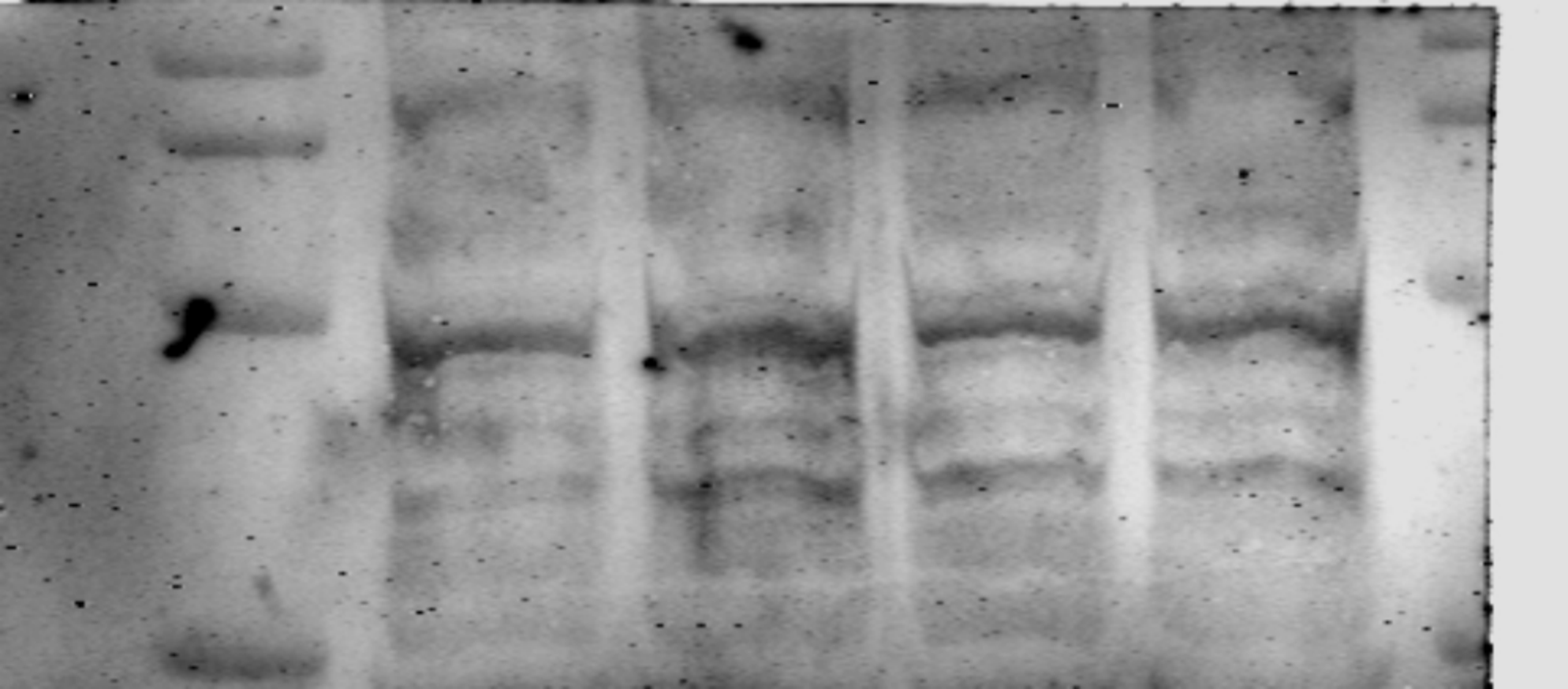

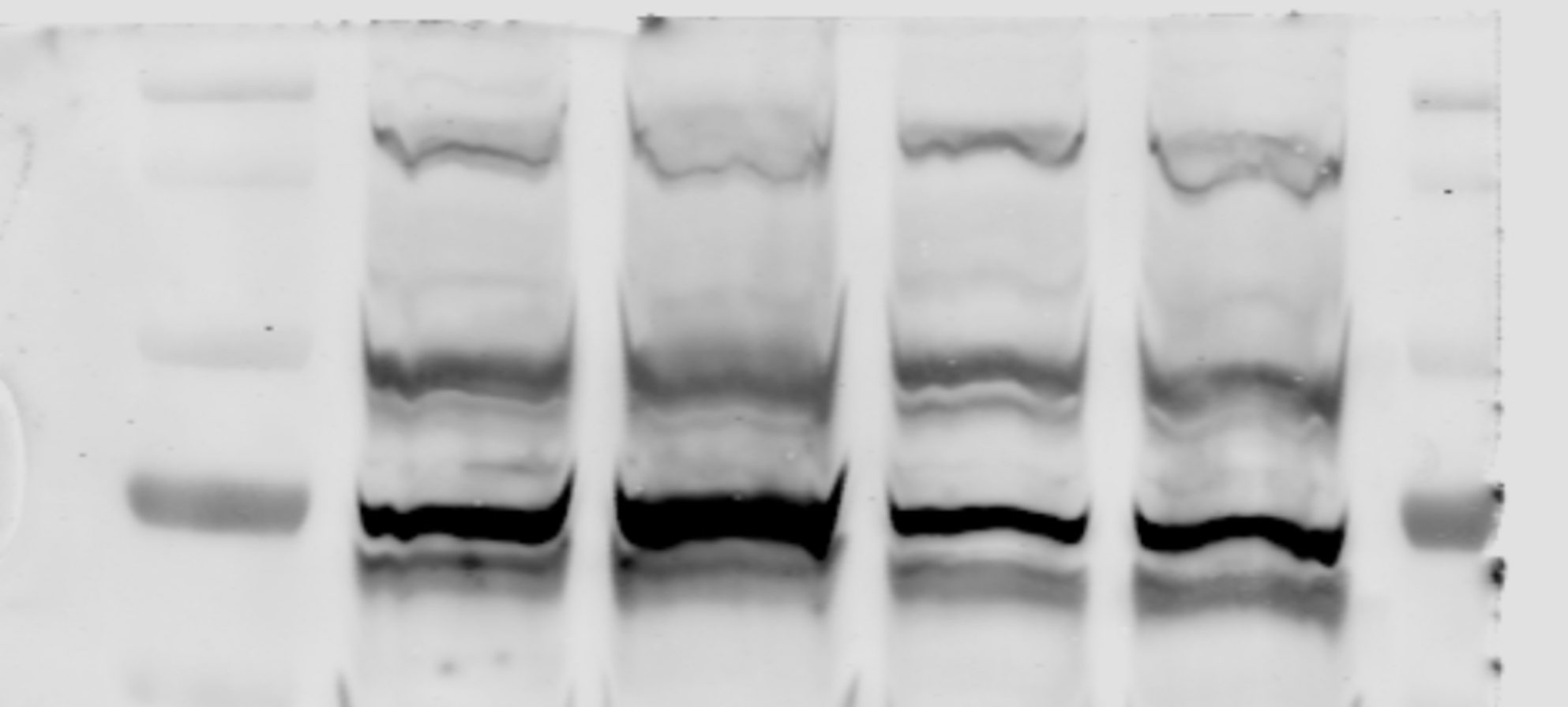

1111

1111

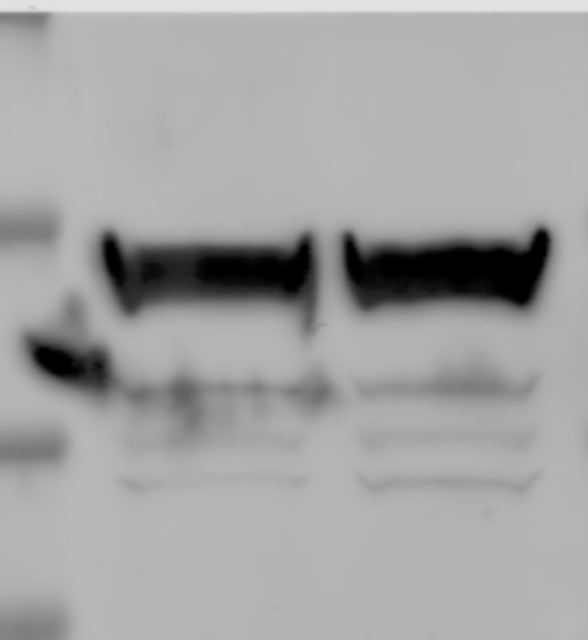

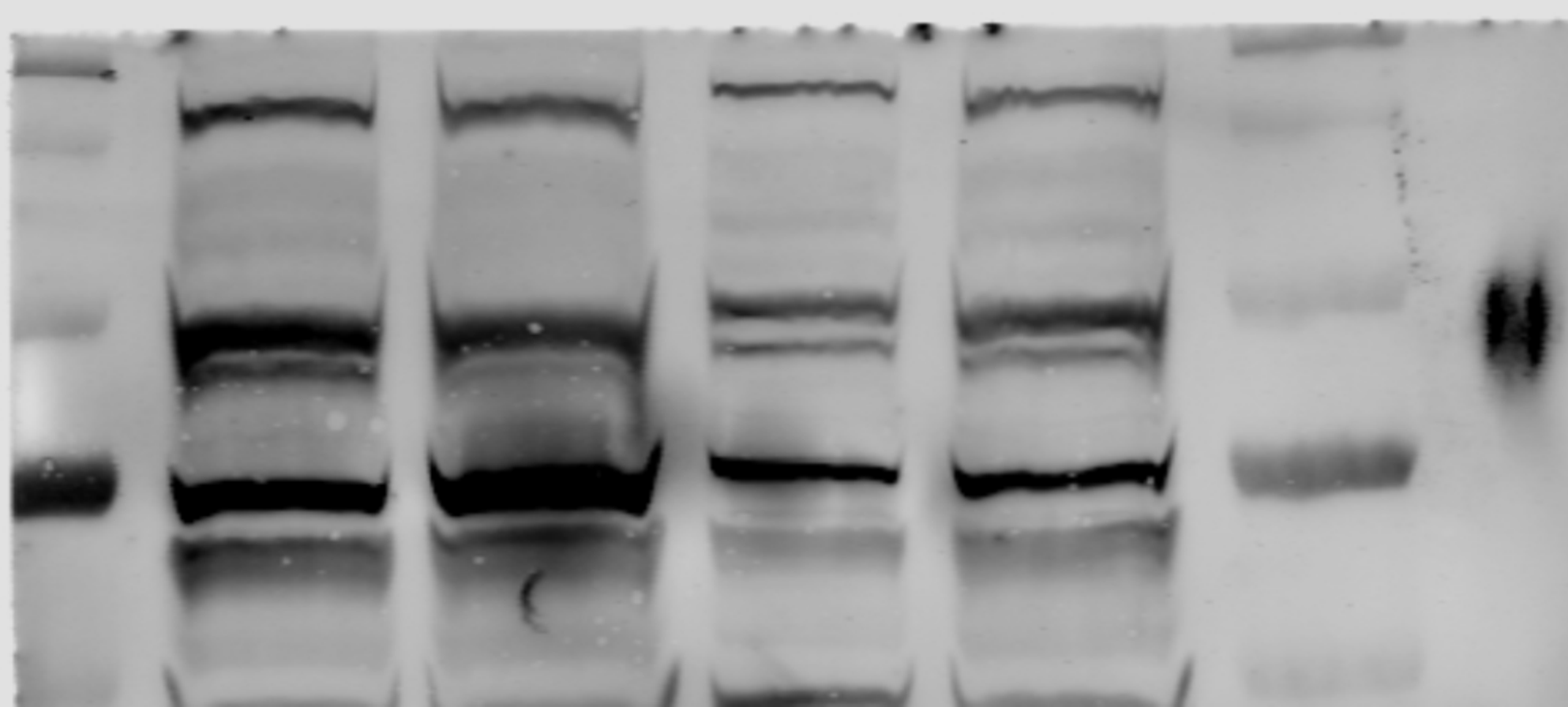

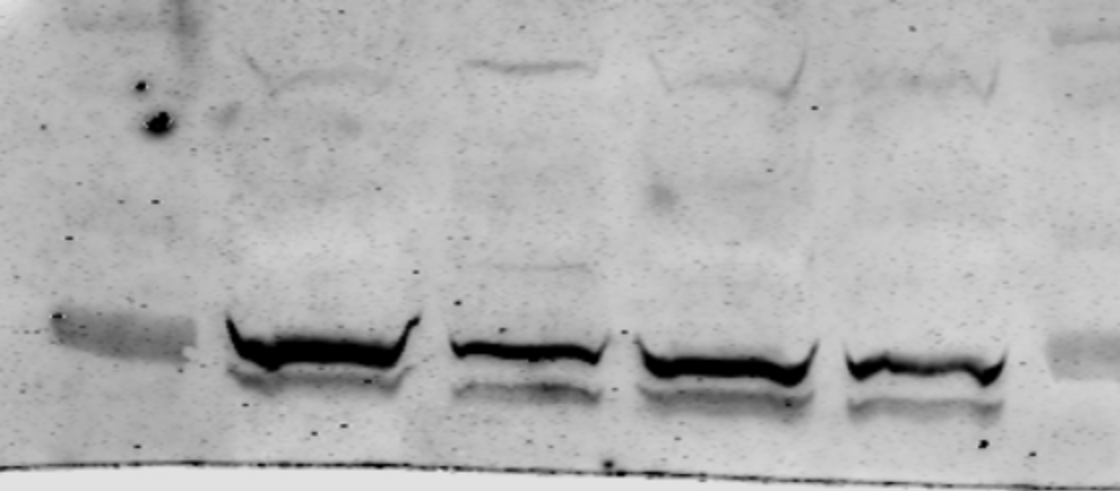

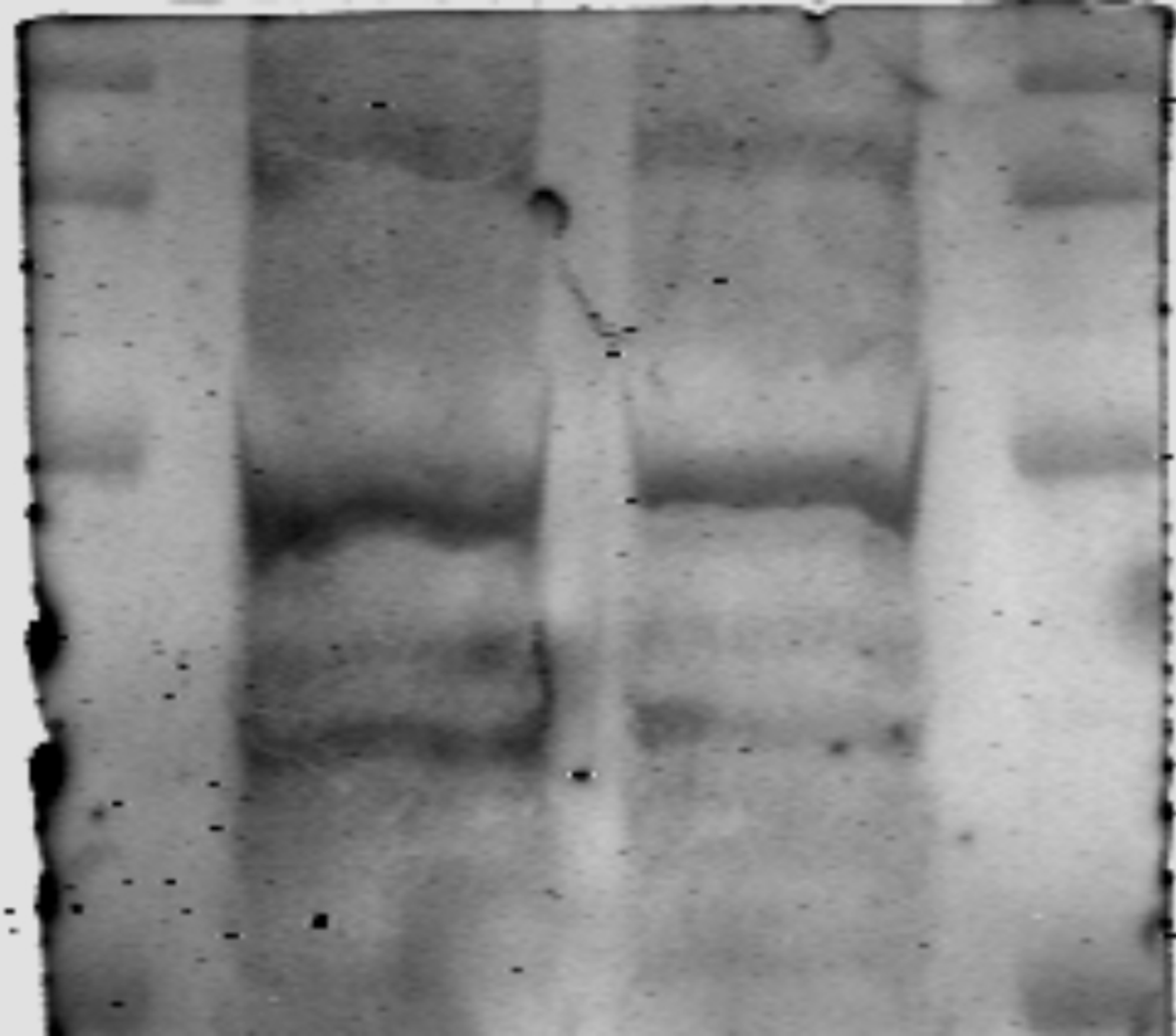

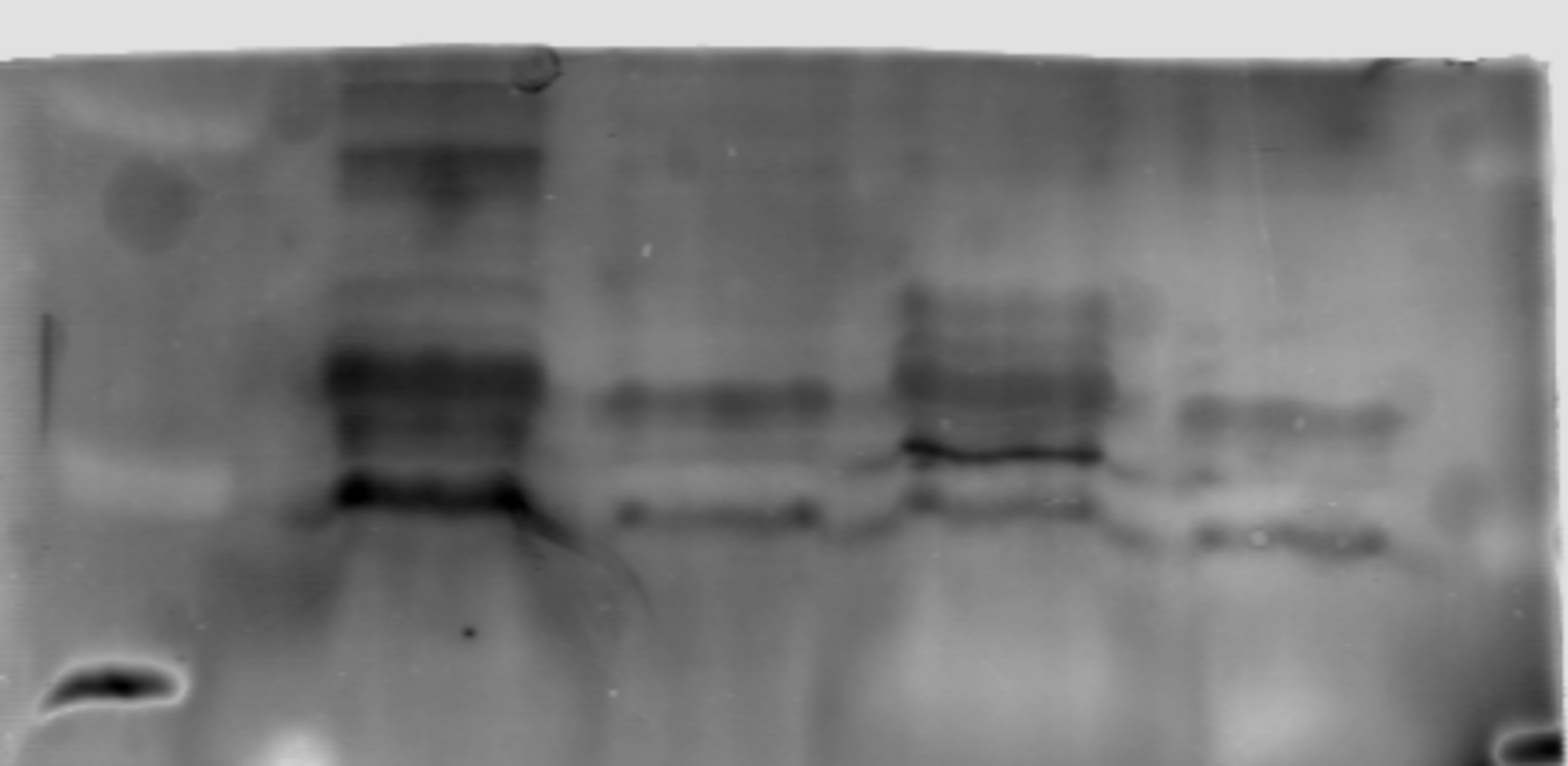

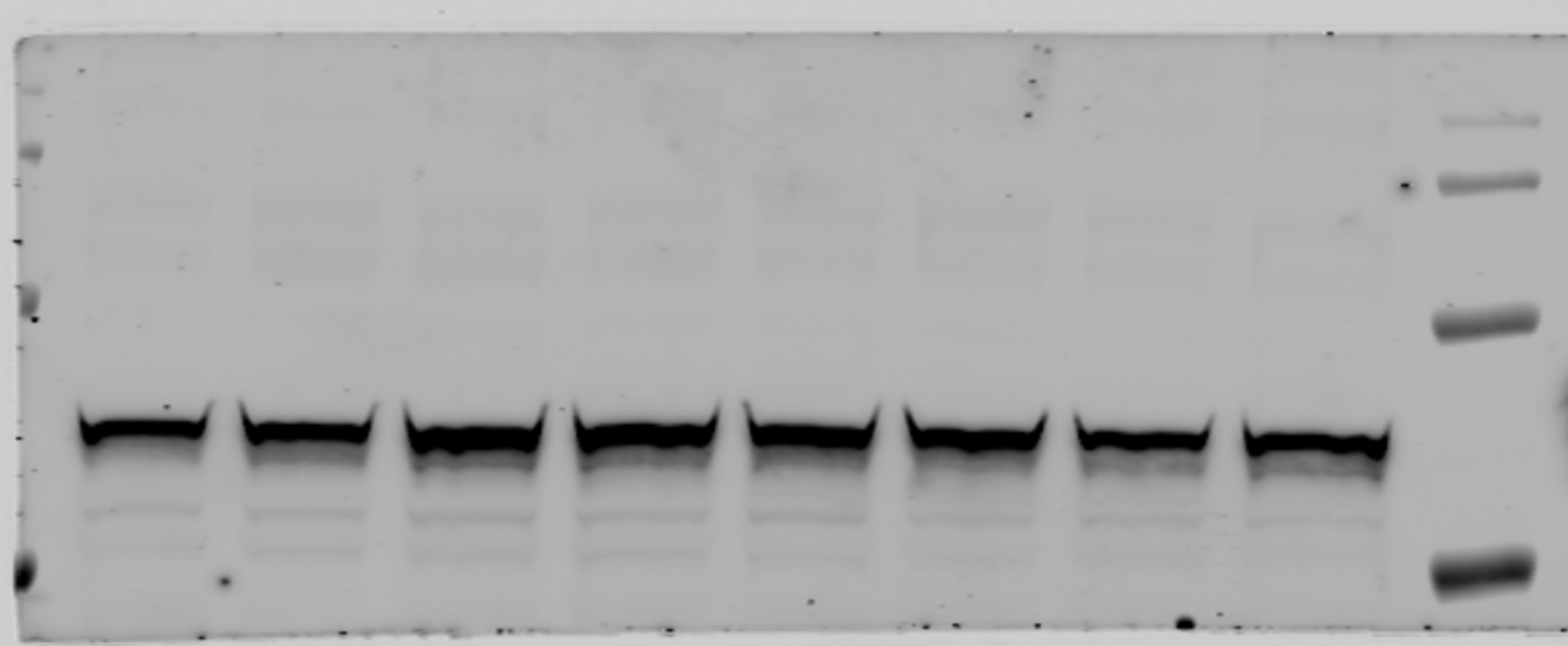

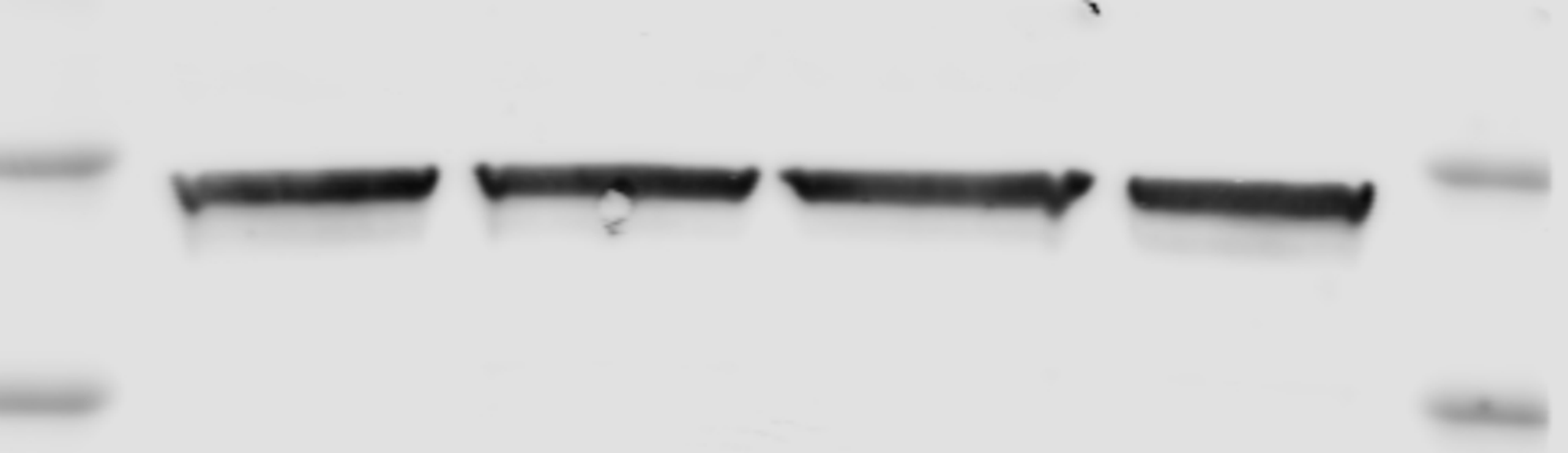

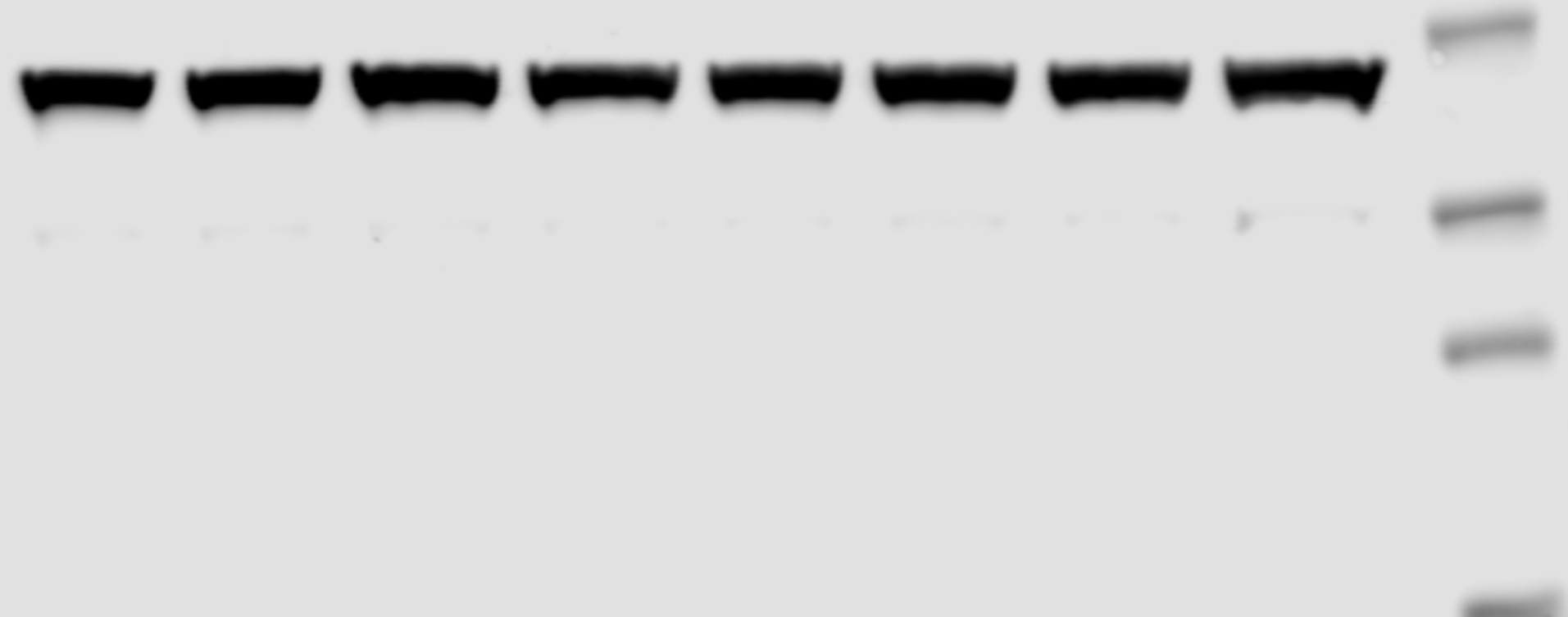

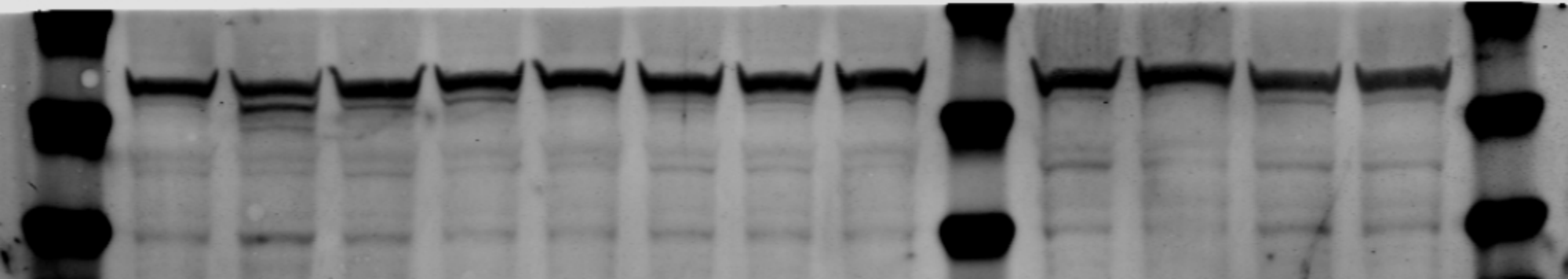

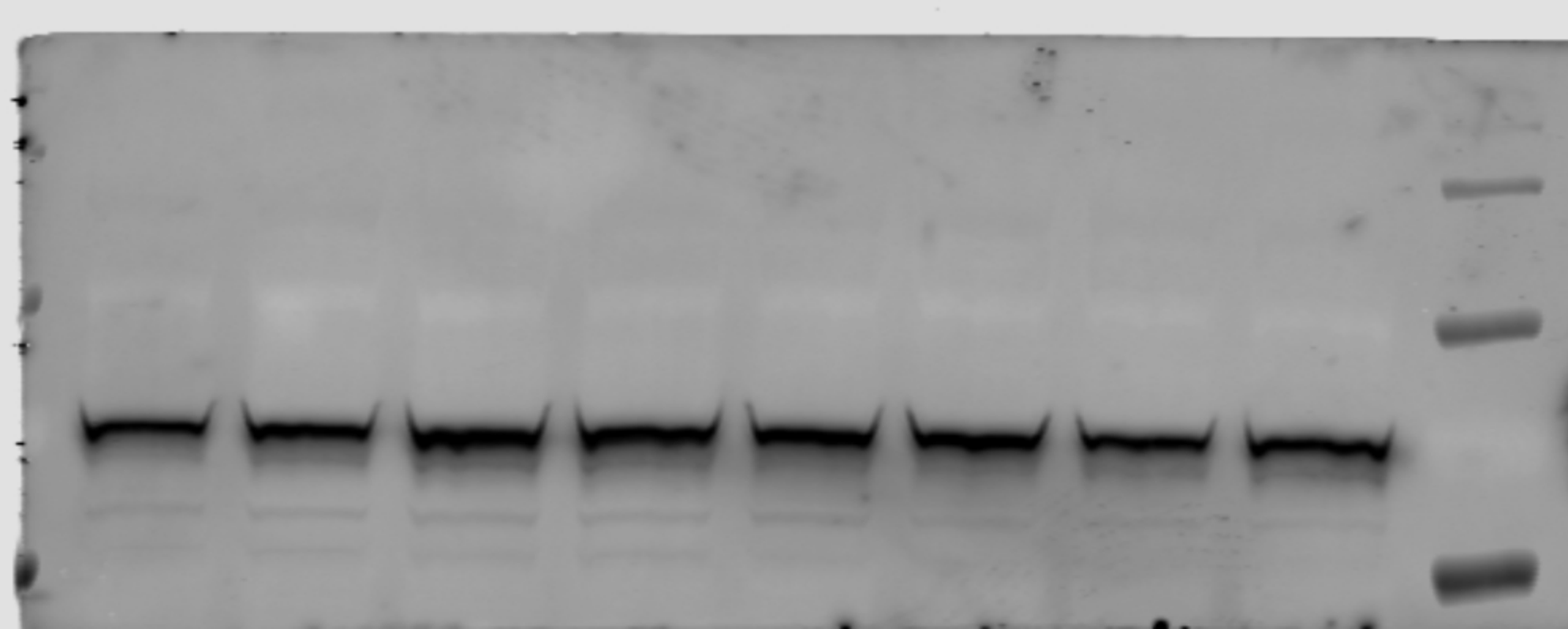

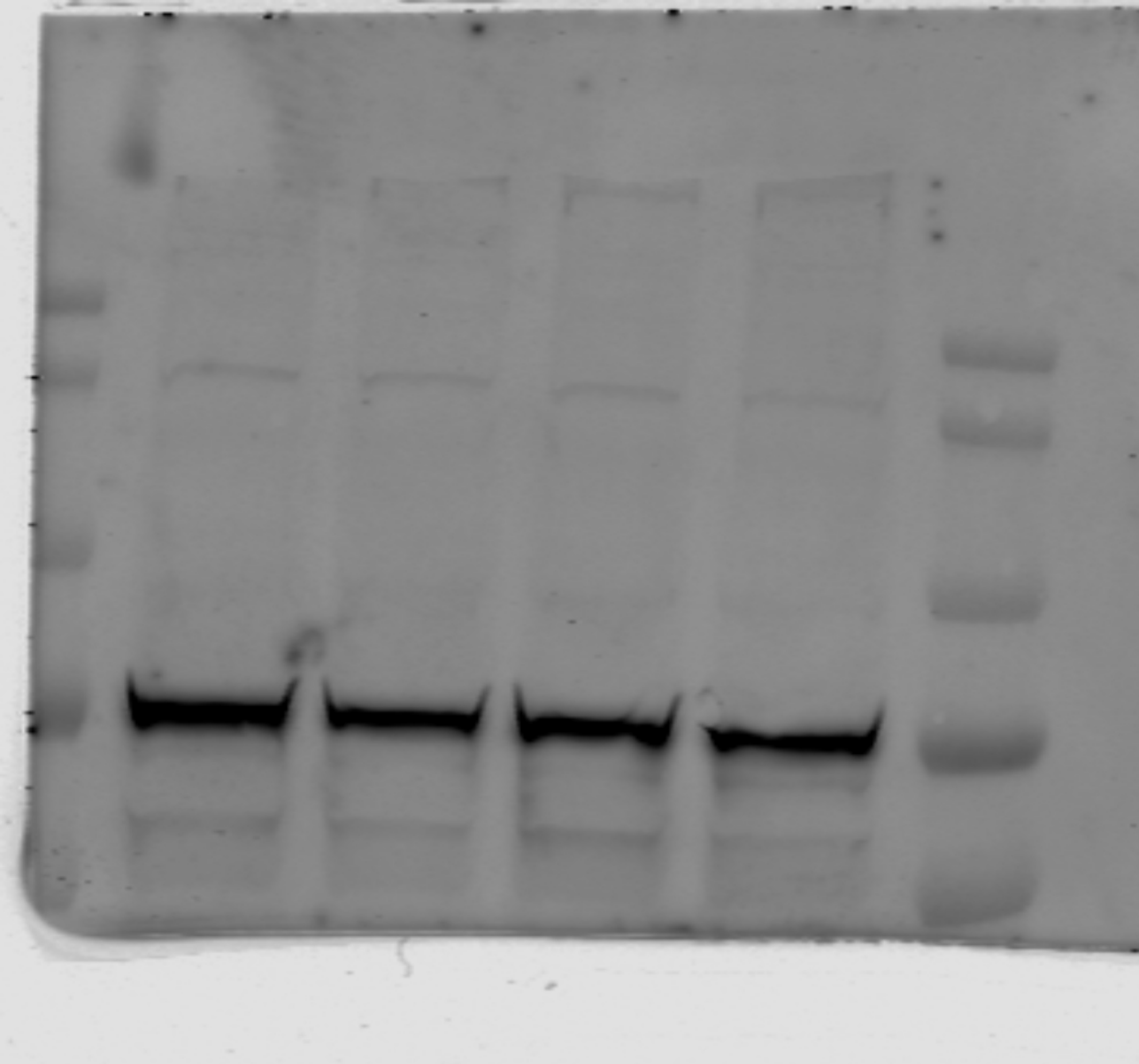

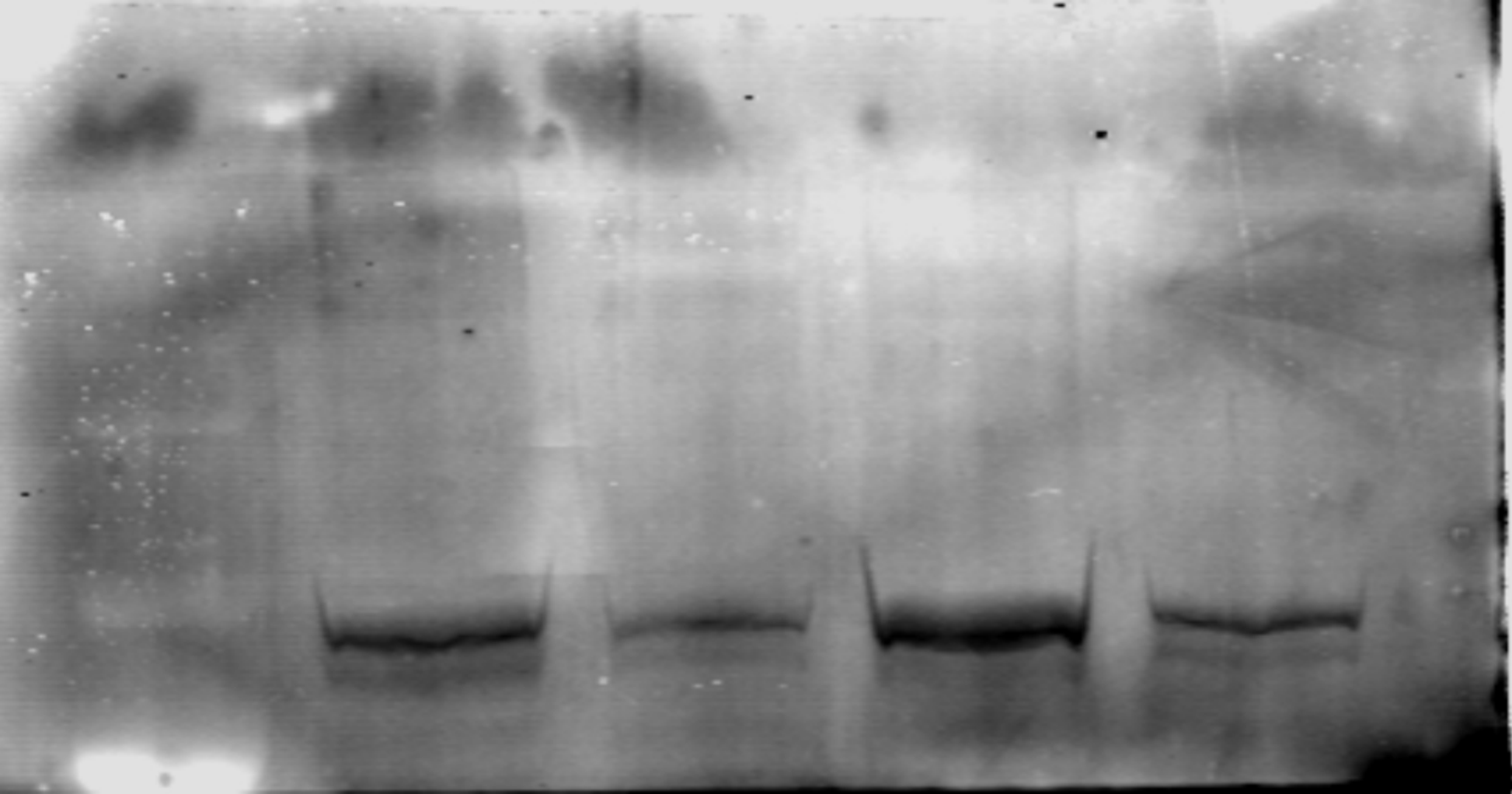

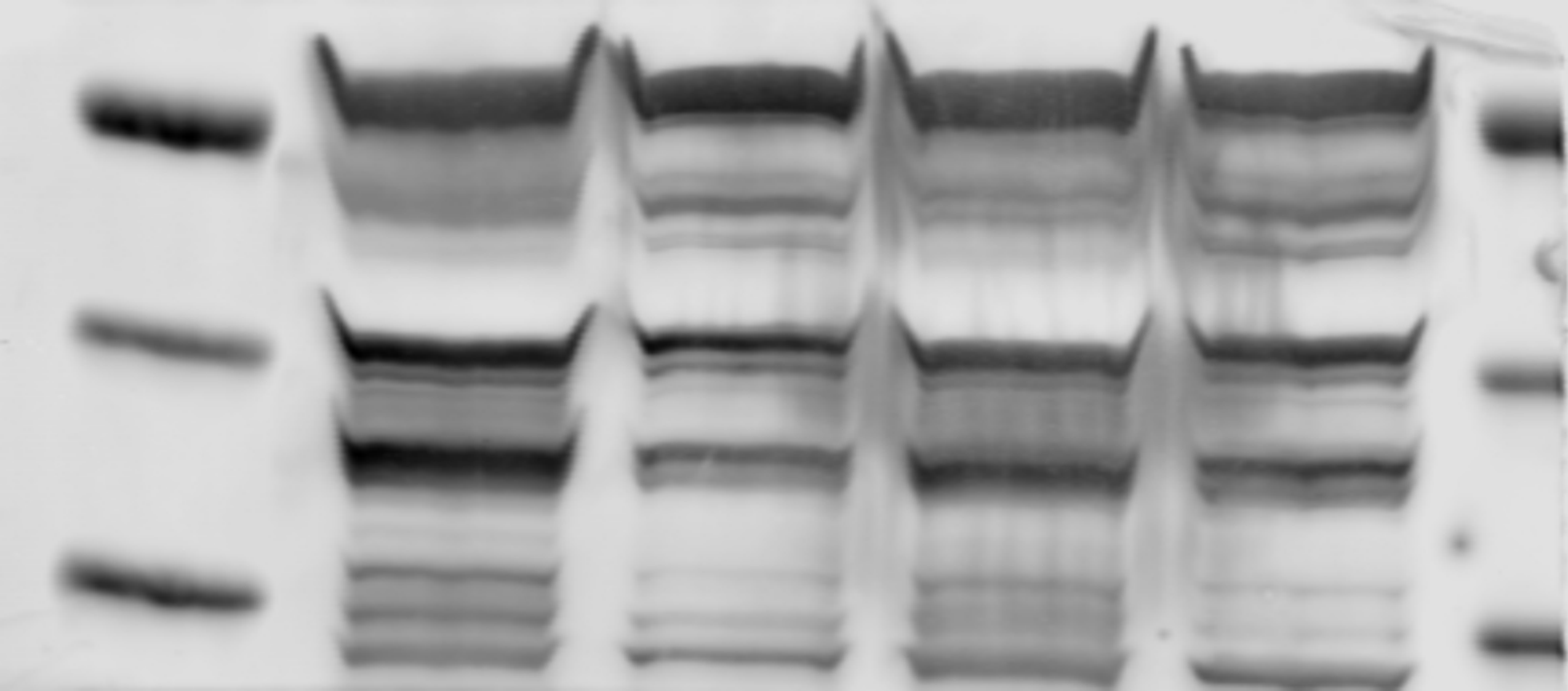

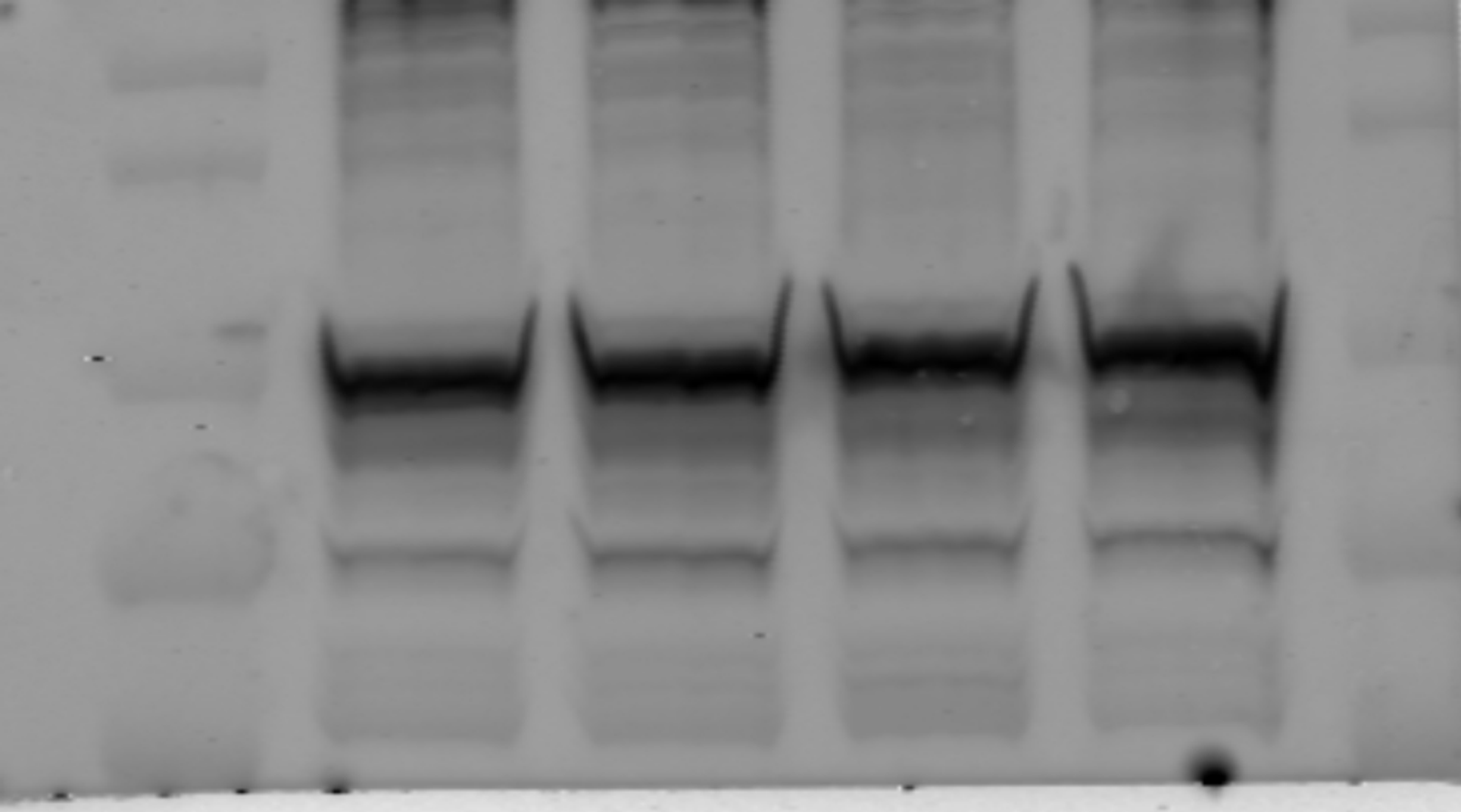

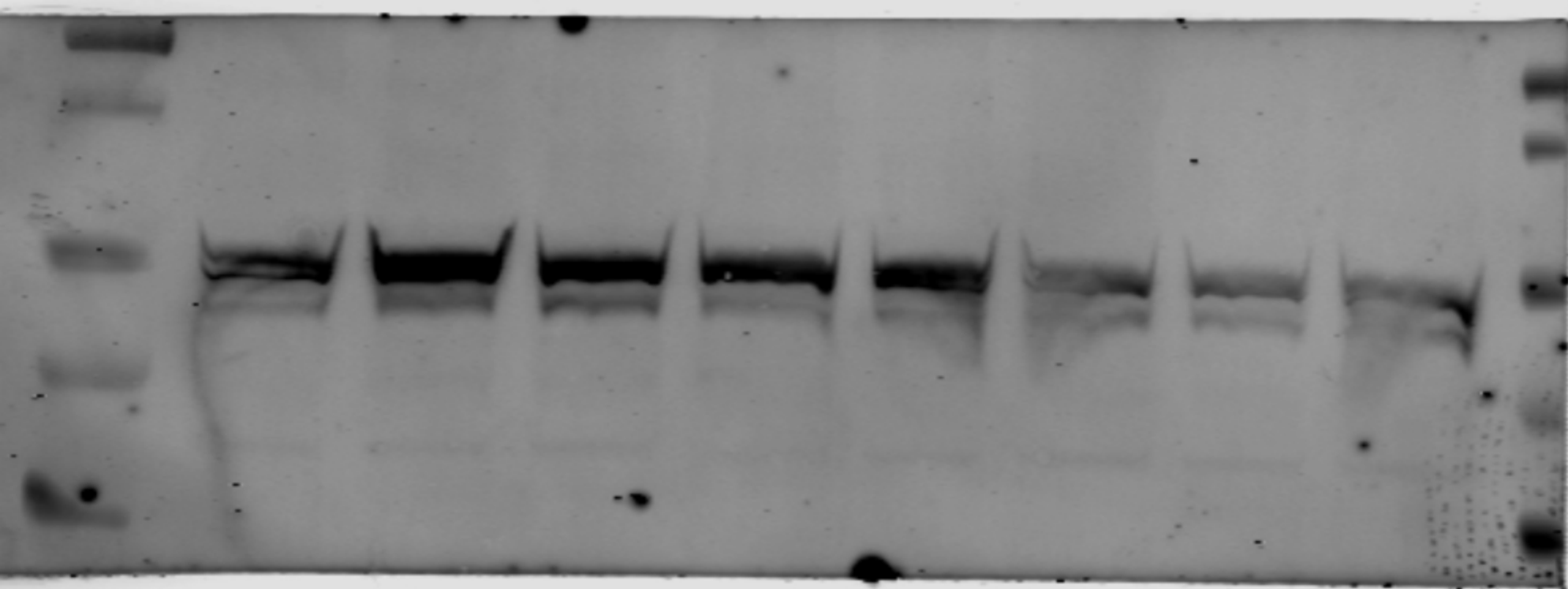

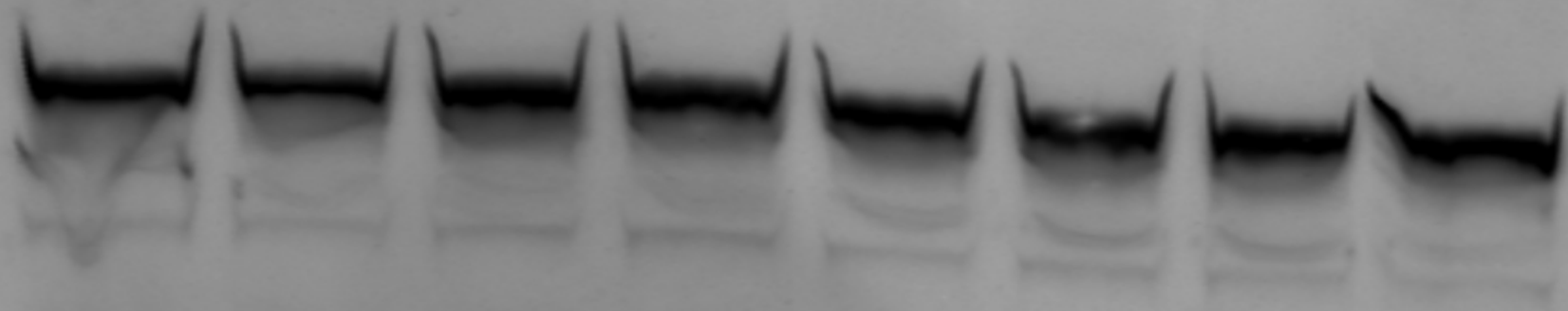

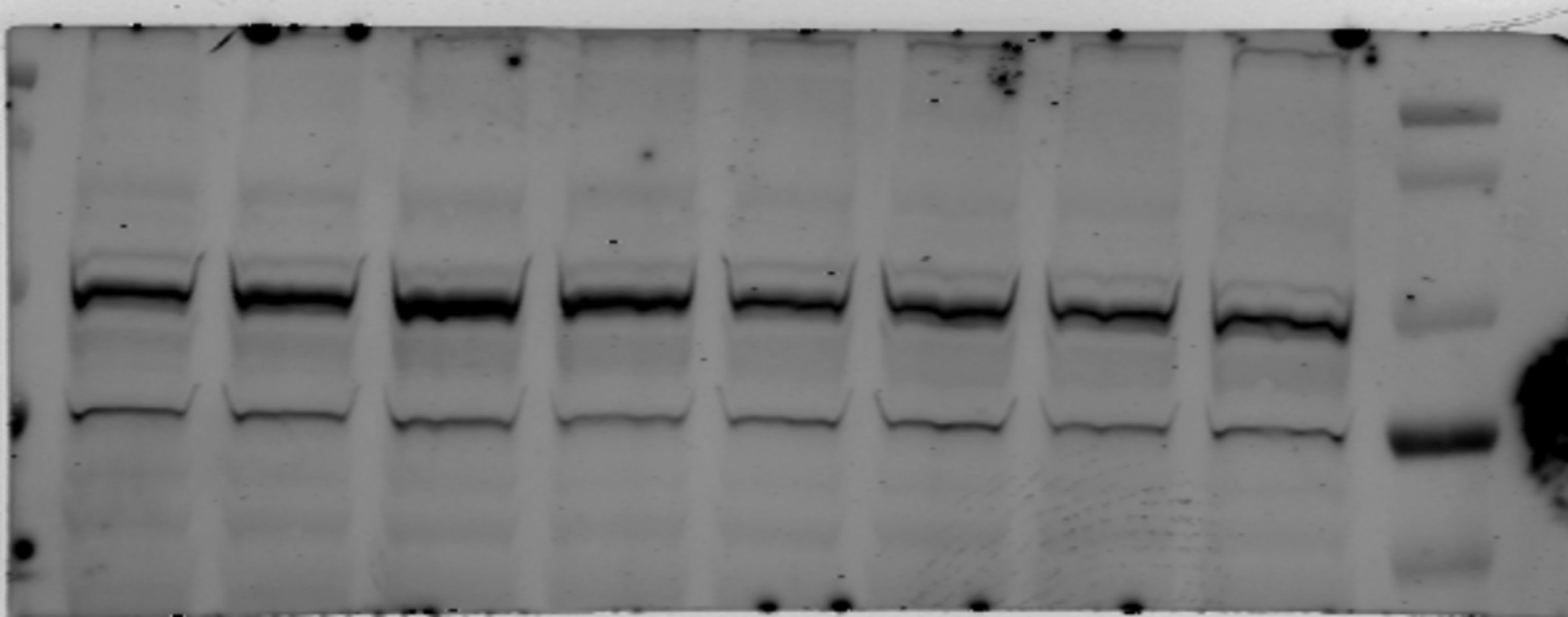

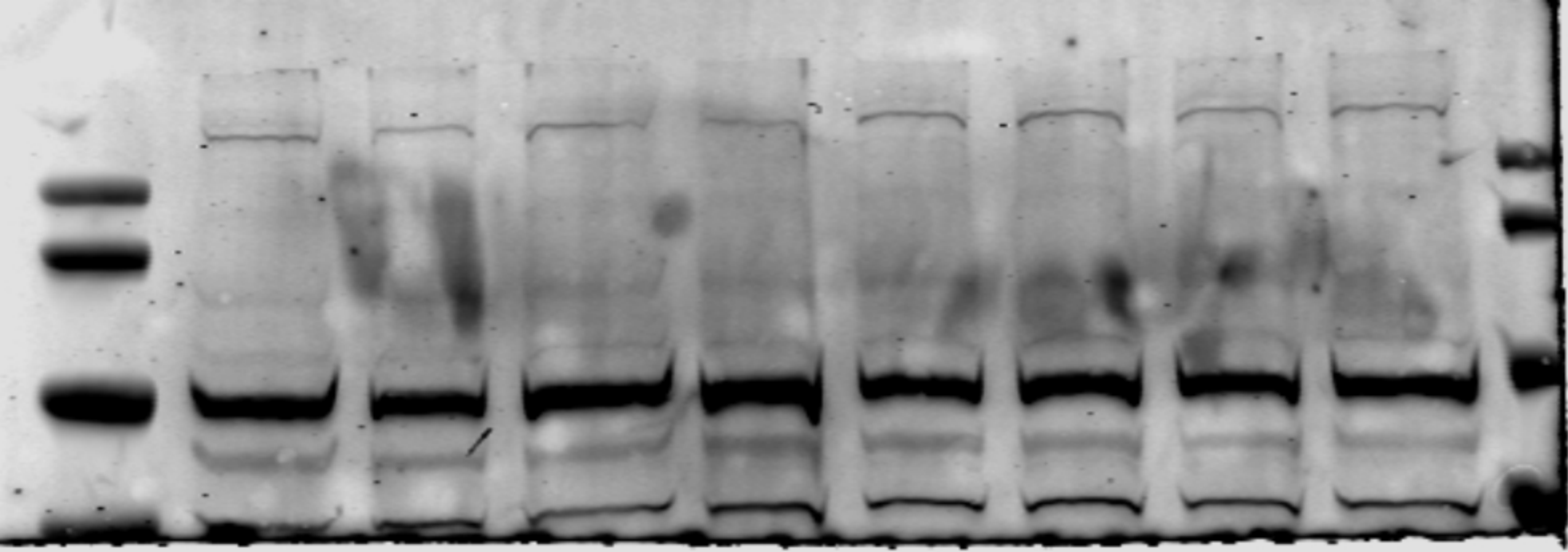

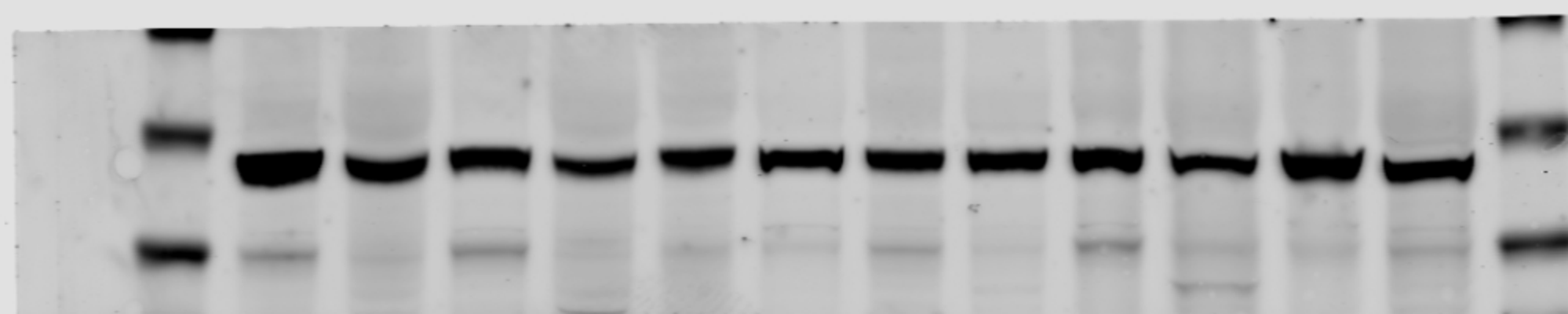

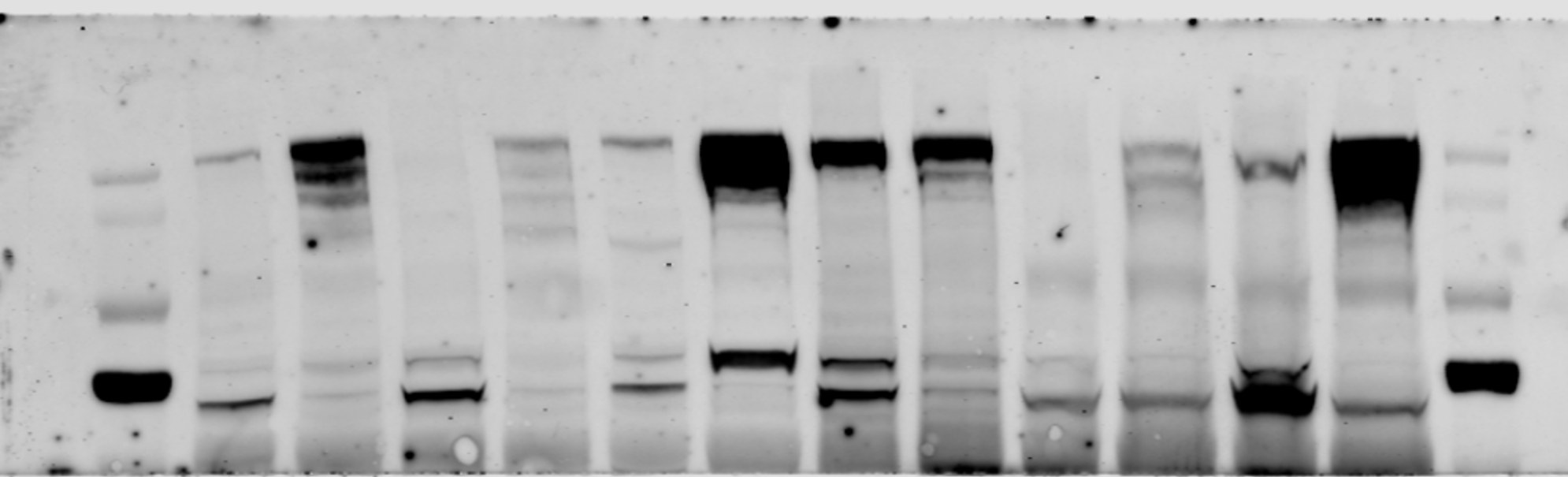

Supplement: Supplementary file 1 — Western blot image [file 41420_2024_1995_MOESM1_ESM.pdf]

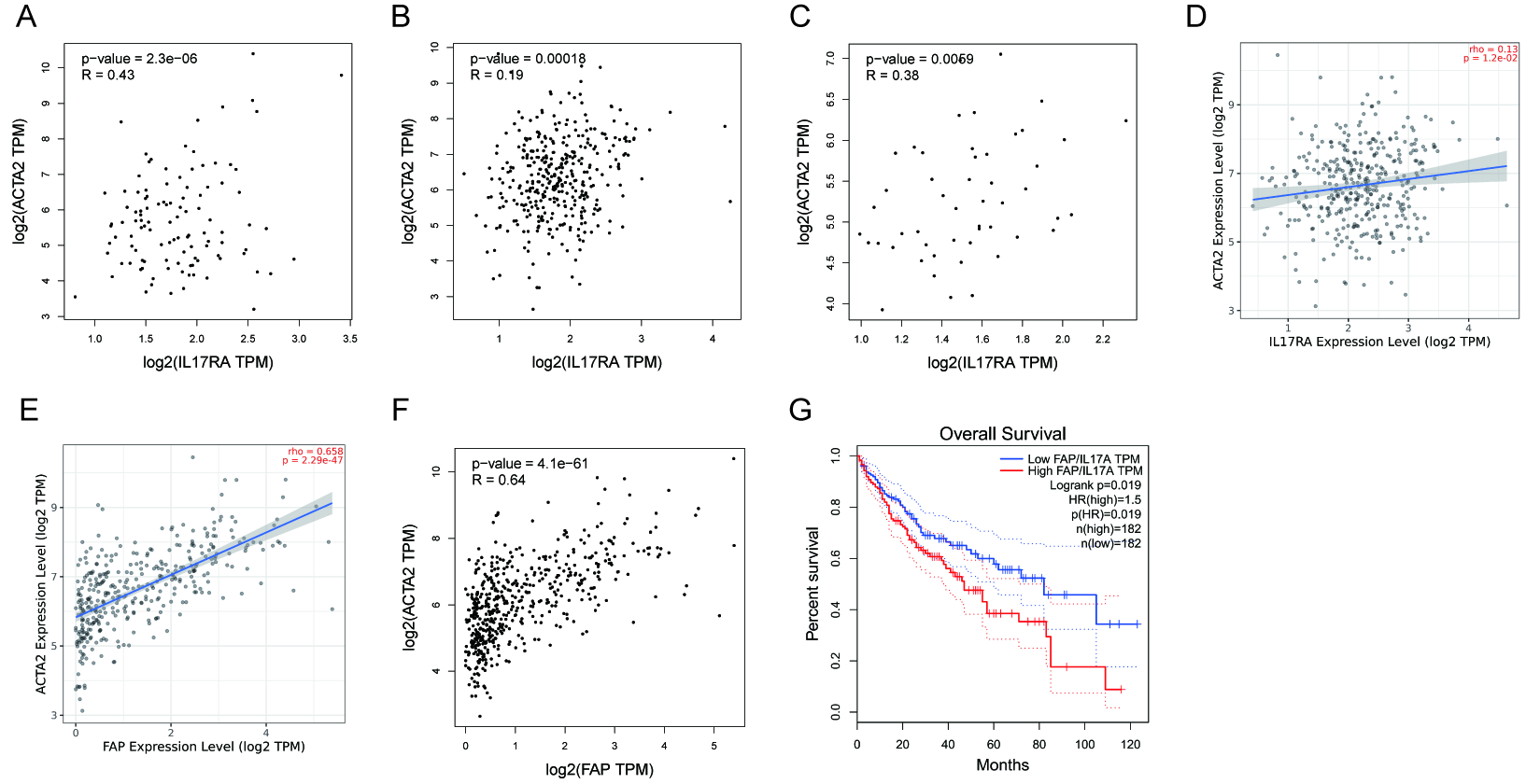

Supplement: Supplementary file 2 — Supplementary Figure 1 [file 41420_2024_1995_MOESM2_ESM.tif]

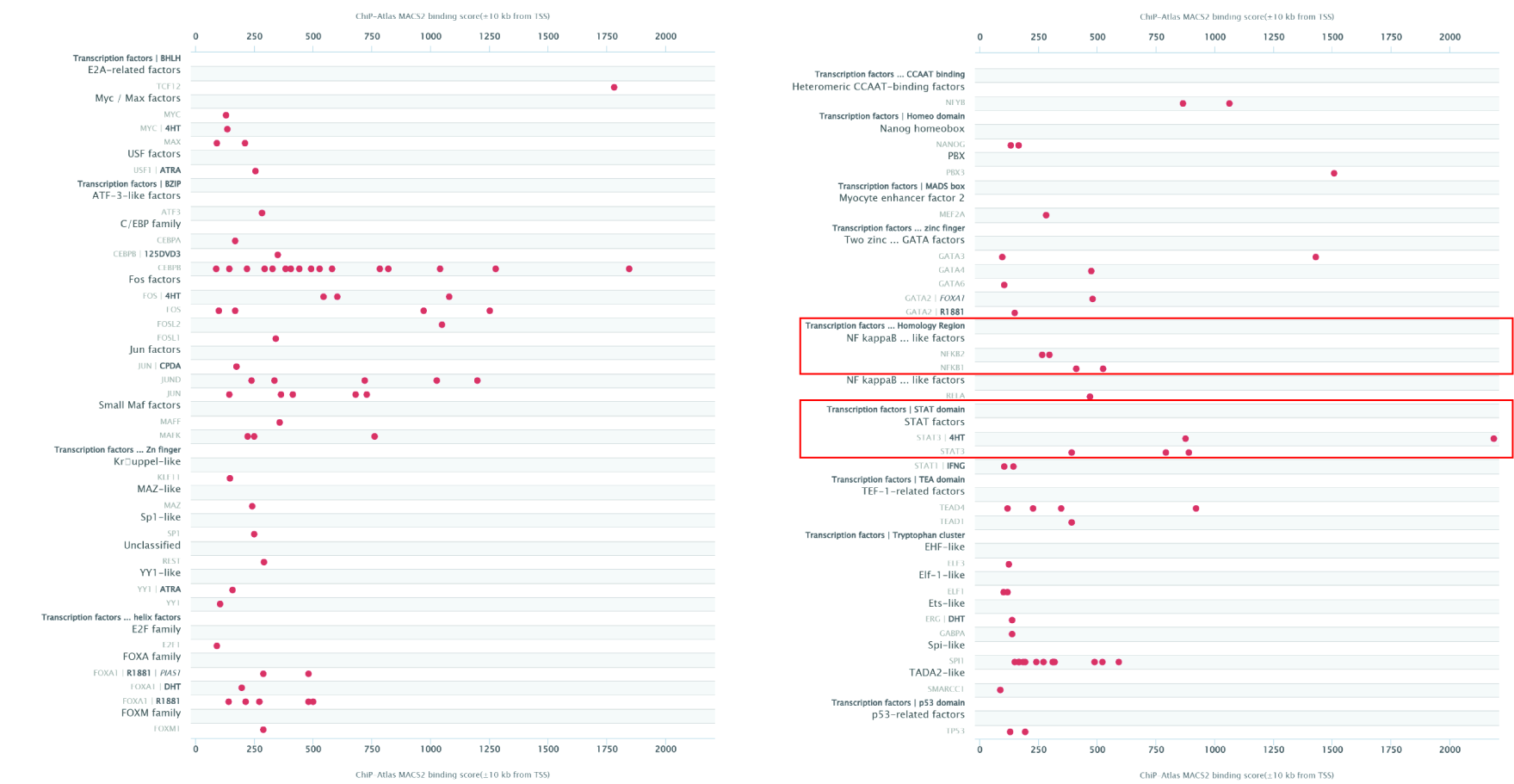

Supplement: Supplementary file 3 — Supplementary Figure 2 [file 41420_2024_1995_MOESM3_ESM.tif]
