# Supplementary material for: IL-17a promotes hepatocellular carcinoma by increasing FAP expression in hepatic stellate cells via activation of the STAT3 signaling pathway
Source: Cell Death Discov. 2024 May 13;10:230. doi: 10.1038/s41420-024-01995-4 (PMC11091202; doi:10.1038/s41420-024-01995-4)
Supplement: Supplementary file 4 — Supplementary information [file 41420_2024_1995_MOESM4_ESM.docx]

**Supplementary Figure 1. Correlations among IL-17RA, FAP and ACTA2 and survival analysis of patients stratified by FAP/IL-17A expression in different public databases.** A. Correlation analysis of IL-17RA and ACTA2 of GTEx liver cohort in the GEPIA database; r=0.43, P=2.3e-06. B. Correlation analysis of IL-17RA and ACTA2 of LIHC Tumor cohort in the GEPIA database; r=0.19, P=0.00018. C. Correlation analysis of IL-17RA and ACTA2 of LIHC Normal cohort in the GEPIA database; r=0.38, P=0.0059. D. Correlation analysis of IL-17RA and ACTA2 in TIMER 2.0; r=0.13, P=1.2e-02. E. Correlation analysis of FAP and ACTA2 in the GEPIA database; r=0.64, P=4.1e-61. F. Correlation analysis of FAP and ACTA2 in TIMER 2.0; r=0.64, P=4.1e-61. G. Overall survival curve of patients stratified by FAP/IL-17A expression in the GEPIA database, indicating poorer survival with higher FAP/IL-17A expression (P=0.019).

**Supplementary Figure 2. The transcription factors of FAP predicted by website.** The various transcription factors bound to the FAP promoter region were predicted by Chip Atlas website. Circled in red are the most common transcription factors.

**Supplementary Table 1.** Proportion of tumor stem cells calculated by ELDA software in subcutaneous tumor tissues of nude mice with tumor cells mixed with IL-17-OE or IL-17-NC cell injected

| **Number of cells injected per mouse** | **Number of tumors** | |
| --- | --- | --- |
|  | FAP-OE | FAP-NC |
| 100000 | 4/6 | 2/6 |
| 500000 | 5/6 | 4/6 |
| 1000000 | 6/6 | 5/6 |
| 5000000 | 6/6 | 5/6 |
| repopulating frequency (95%CI) | 1/176232 (1/387177,1/80216) | 1/941069 (1/1930980,1/458633) |
| P value | 0.000386 | |

**Supplementary Table 2.** Proportion of tumor stem cells calculated by ELDA software in subcutaneous tumor tissues of nude mice with tumor cells mixed with IL-17-OE or IL-17-NC cell injected

| **Number of cells injected per mouse** | **Number of tumors** | |
| --- | --- | --- |
|  | IL-17-OE | IL-17-NC |
| 100000 | 4/6 | 2/6 |
| 500000 | 5/6 | 3/6 |
| 1000000 | 6/6 | 4/6 |
| 5000000 | 6/6 | 5/6 |
| repopulating frequency (95%CI) | 1/176232 (1/387177,1/80216) | 1/1057814 (1/2178096,1/513738) |
| P value | 0.000168 | |

**Supplementary Table 3.** The predicted binding sites of transcription factor STAT3 to FAP promoter region

| **Name** | **Score** | **Relative score** | **Start** | **End** | **Strand** | **Predicted sequence** |
| --- | --- | --- | --- | --- | --- | --- |
| STAT3 | 9.926147 | 0.917805582 | 1332 | 1342 | + | CTGCTGGTAAA |
| STAT3 | 9.8443575 | 0.916814733 | 450 | 460 | + | CTTCTGAGAAC |
| STAT3 | 7.0655336 | 0.883150521 | 697 | 707 | + | CTGCCAGTAAG |
| STAT3 | 6.0610943 | 0.870982186 | 1939 | 1949 | + | TTTACAGAAAT |
| STAT3 | 5.9303 | 0.869397674 | 39 | 49 | + | TTTCTTAAAAC |
| STAT3 | 5.6520476 | 0.866026768 | 101 | 111 | + | TTCCAAGGAAA |
| STAT3 | 5.090459 | 0.859223371 | 780 | 790 | + | CTGAAAGGAAG |
